# Fluorescent protein-tagged Vpr dissociates from HIV-1 core after viral fusion and rapidly enters the cell nucleus

**DOI:** 10.1186/s12977-015-0215-z

**Published:** 2015-10-29

**Authors:** Tanay M. Desai, Mariana Marin, Chetan Sood, Jiong Shi, Fatima Nawaz, Christopher Aiken, Gregory B. Melikyan

**Affiliations:** Division of Pediatric Infectious Diseases, Emory University School of Medicine, Atlanta, GA USA; Children’s Healthcare of Atlanta, Atlanta, GA USA; Department of Pathology, Microbiology and Immunology, Vanderbilt University, Nashville, TN 37232 USA

**Keywords:** Viral fusion, Single particle tracking, Fluorescence correlation spectroscopy, Confocal imaging, Beta-lactamase assay, HIV capsid mutant, Vpr, FRAP, Nuclear transport

## Abstract

**Background:**

HIV-1 Vpr is recruited into virions during assembly and appears to remain associated with the viral core after the reverse transcription and uncoating steps of entry. This feature has prompted the use of fluorescently labeled Vpr to visualize viral particles and to follow trafficking of post-fusion HIV-1 cores in the cytoplasm.

**Results:**

Here, we tracked single pseudovirus entry and fusion and observed that fluorescently tagged Vpr gradually dissociates from post-fusion viral cores over the course of several minutes and accumulates in the nucleus. Kinetics measurements showed that fluorescent Vpr released from the cores very rapidly entered the cell nucleus. More than 10,000 Vpr molecules can be delivered into the cell nucleus within 45 min of infection by HIV-1 particles pseudotyped with the avian sarcoma and leukosis virus envelope glycoprotein. The fraction of Vpr from cell-bound viruses that accumulated in the nucleus was proportional to the extent of virus-cell fusion and was fully blocked by viral fusion inhibitors. Entry of virus-derived Vpr into the nucleus occurred independently of envelope glycoproteins or target cells. Fluorescence correlation spectroscopy revealed two forms of nuclear Vpr—monomers and very large complexes, likely involving host factors. The kinetics of viral Vpr entering the nucleus after fusion was not affected by point mutations in the capsid protein that alter the stability of the viral core.

**Conclusions:**

The independence of Vpr shedding of capsid stability and its relatively rapid dissociation from post-fusion cores suggest that this process may precede capsid uncoating, which appears to occur on a slower time scale. Our results thus demonstrate that a bulk of fluorescently labeled Vpr incorporated into HIV-1 particles is released shortly after fusion. Future studies will address the question whether the quick and efficient nuclear delivery of Vpr derived from incoming viruses can regulate subsequent steps of HIV-1 infection.

**Electronic supplementary material:**

The online version of this article (doi:10.1186/s12977-015-0215-z) contains supplementary material, which is available to authorized users.

## Background

HIV-1 infection is initiated through fusion of viral and cellular membranes which leads to the release of nucleocapsid into the cytoplasm. Incoming nucleocapsids reverse transcribe their RNA genome and undergo uncoating, which is manifested in, at least partial, shedding of structural proteins, such as capsid and matrix (reviewed in [1]). The remaining nucleoprotein complex, referred to as a pre-integration complex (PIC), enters the nucleus and integrates viral DNA into the host genome. In spite of extensive efforts, the early post-fusion steps of HIV-1 entry, uncoating and nuclear import, are poorly understood. Single virus imaging is a relatively new approach which, unlike the biochemical assays reporting an ensemble averaged behavior of incoming capsids, enables the visualization of the fate of individual post-fusion particles in living cells. By incorporating fluorescently labeled proteins into HIV-1 particles, researchers have been able to visualize the fusion, uncoating and reverse transcription steps, as well as subsequent entry of PICs into the nucleus [2–10].

A widely used means to incorporate proteins, including GFP derivatives, into HIV-1 particles is through chimeras with the viral accessory protein Vpr, which is recruited into virions through interactions with the p6 domain of Gag [11, 12]. This strategy has been successfully used to incorporate integrase and reverse transcriptase, as well as cyclophilin A and β-lactamase, into virions [13–16]. Given that a relatively large number of Vpr molecules (300 or more copies [17–19]) can be incorporated into HIV-1 particles, Vpr fusions with fluorescent proteins have proven useful for visualization of single virus and single core trafficking in the cytoplasm [2, 20–25]. In these imaging studies, pseudoviruses co-labeled with GFP-Vpr and a membrane marker have been used to identify post-fusion viral cores based upon separation of the two markers following virus fusion with the cell membrane. However, reliable identification of post-fusion cores using this strategy critically relies on: (1) the presence of both core and membrane markers in all HIV-1 particles and (2) spatial separation of the capsid and the viral membrane, which may be delayed relative to the fusion event and/or occur inefficiently. To our knowledge, the fate of post-fusion cores has not been examined, using more direct techniques that enable the visualization of viral fusion based upon the release of a viral content marker (see for example [26]).

Here, we employed time-resolved single particle imaging to visualize both the viral fusion events (detected as release of the viral content marker, mCherry [23, 26]) and the fate of post-fusion viral cores labeled with YFP-Vpr or GFP-Vpr. We observed that, surprisingly, all the particles that fused (rapid loss of mCherry), gradually lost YFP-Vpr signal over several minutes, thereafter. Loss of punctate YFP-Vpr signal was associated with concomitant appearance of diffuse YFP fluorescence in the cell nucleus. Fluorescence correlation spectroscopy showed that fluorescent Vpr in the nucleus existed as a monomer and as a part of very large cellular complexes. Nuclear accumulation of labeled Vpr correlated with the virus fusion activity. We found that the extent of nuclear entry of YFP-Vpr varied between viral preparations, but appeared independent of the capsid stability. These findings show that Vpr, at least partially, dissociates from the post-fusion HIV-1 cores and quickly enters the nucleus. Interestingly, and in stark contrast to live cell experiments, detergent lysis of immobilized viruses in vitro, in the presence or in the absence of cytosol, did not promote loss of YFP-Vpr. Stability of YFP-Vpr signal upon virus lysis in vitro suggests that active processes occurring in the cytoplasm promote shedding of this marker from the incoming viral cores.

## Results

### Fluorescently tagged Vpr is released from virions after fusion

We sought to visualize the fate of individual HIV-1 cores after viral fusion using pseudoviruses co-labeled with the fluorescently tagged Vpr protein (YFP-Vpr or GFP-Vpr) in the core and the viral content marker Gag-imCherry [23, 26]. The Gag-imCherry chimera contains an “internal” mCherry inserted between the MA and CA sequences of Gag polyprotein. Cleavage of Gag-imCherry upon virus maturation produces free mCherry that is released from viral particles as a result of fusion [26]. Thus, loss of mCherry marks the point of fusion, while YFP-Vpr serves as a reference signal to track single particles. Efficient fusion is accomplished through pseudotyping the HIV-1 core either with the avian sarcoma and leukosis virus (ASLV) Env [26, 27] or the vesicular stomatitis virus G glycoprotein (VSV-G).

**Fig. 1:**
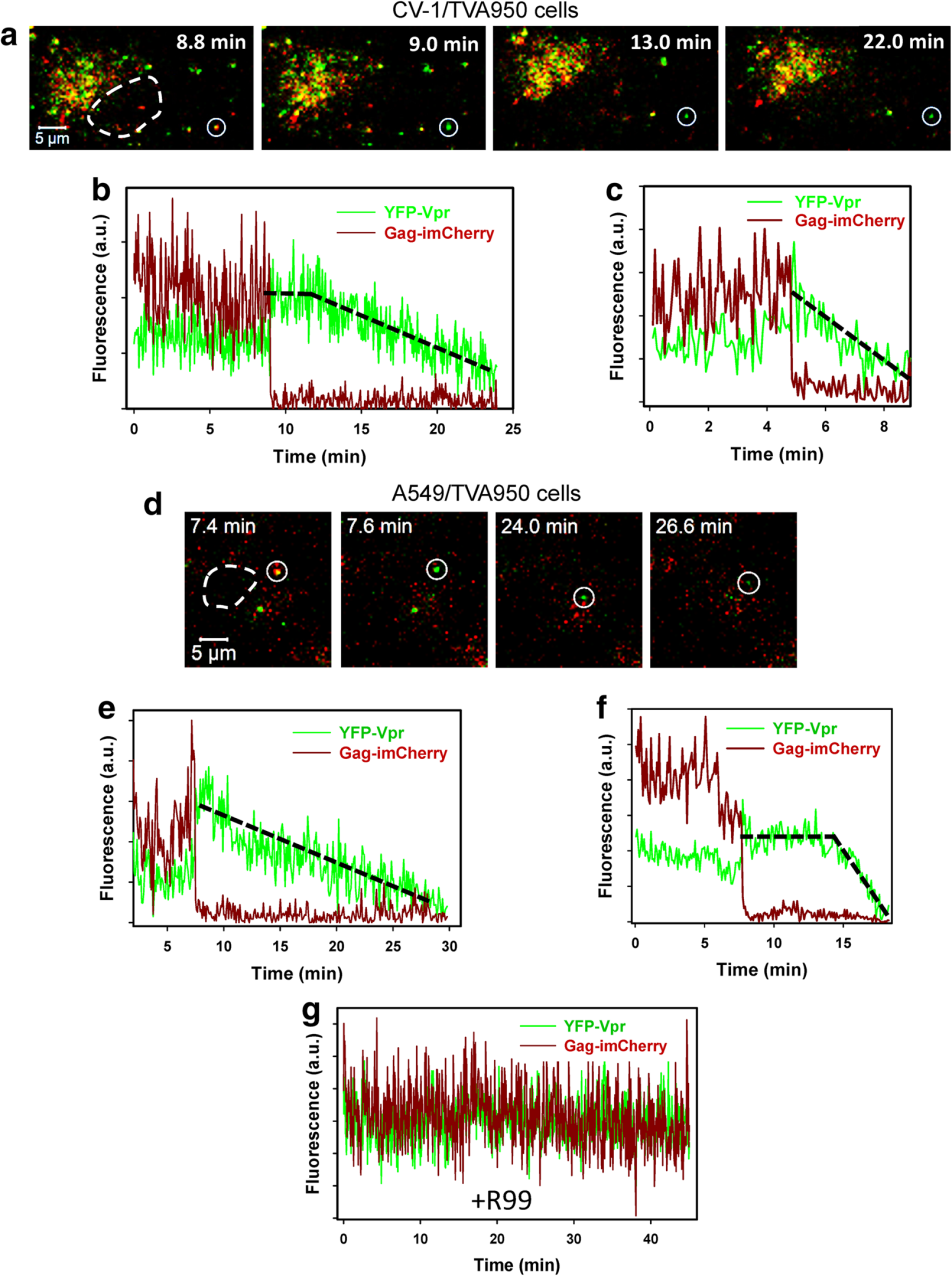
Post-fusion decay of HIV-1 YFP-Vpr signal. **a**, **d** ASLVpp co-labeled with the core-associated YFP-Vpr (*green*) and a releasable content marker Gag-imCherry (*dark red*) were pre-bound in the cold to CV-1/TVA950 (**a**–**c**, **g**) or A549/TVA950 (**d**–**f**) cells expressing the ASLV receptor TVA950. Entry was initiated by introducing warm buffer, and cells were maintained at 37 °C for 45 min and imaged every 3–5 s. Fusing viruses were detected by the near-instantaneous disappearance of mCherry from double-labeled particles (marked by *white circles* in **a** and **d**). *White dashed lines* show the boundaries of cell nuclei. **b**, **c** Fluorescence intensity profiles (total fluorescence of YFP-Vpr and Gag-imCherry) obtained by single ASLVpp tracking in CV-1-derived cells. **e**, **f** Fluorescence intensity profiles for YFP-Vpr and Gag-imCherry obtained by single ASLVpp tracking in an A549-derived cell. **g** An example of YFP-Vpr and Gag-imCherry signals from a non-fusing particle selected from an experiment carried out in the presence of the ASLV fusion inhibitor R99 (50 μg/ml). *Black dashed lines* outline different YFP decay profiles occurring without (**c**, **e**) and with a lag (**b**, **f**) after the release of mCherry. Here and in Fig. 2, the abrupt ending of fluorescence traces occurs due to the inability to track faint YFP/GFP-Vpr puncta using particle tracking software, as the signal approaches the background level

Pseudoviruses were pre-bound to permissive cells in the cold, and viral entry/fusion was initiated by shifting to 37 °C for 45 min (unless stated otherwise). Single ASLV Env-pseudotyped particles (ASLVpp) efficiently fused with cells expressing high levels of TVA, the receptor for subtype A virus, as evidenced by an abrupt loss of mCherry and concomitant transient increase of the pH-sensitive YFP fluorescence (Fig. 1a–f). The increase in the YFP signal (pKa ~7.0 [28]) at the time of fusion most likely occurs due to the slightly higher pH in the cytosol compared to the intraviral pH [26]. The overwhelming majority of observable single fusion events occurred within the first 20 min of incubation (see below). At later times, reliable detection of the mCherry release events was precluded by clustering of unfused viruses within the autofluorescent perinuclear area.

Interestingly, the initial increase in the YFP-Vpr signal at the time of fusion with CV-1- or A549-derived cell lines was followed by fluorescence decay over the course of several minutes (Fig. 1a–f). All single ASLVpp that we were able to track in these two cell lines, using tracking software or by visual observation (370 particles total), lost YFP-Vpr within about 15–20 min after fusion (Fig. 1a–f). This characteristic gradual decrease in the YFP signal after fusion has also been observed in our previous study [26]. The loss of YFP-Vpr was not caused by photobleaching, since the mCherry and YFP signals from non-fusing particles did not change considerably throughout the imaging experiments (Fig. 1g). Also, because post-fusion viral cores are expected to reside in the cytosol, acidification of the viral interior as the reason for the vanishing YFP signal can also be ruled out. The YFP-Vpr decay started either immediately (Fig. 1c, e) or several minutes after the release of mCherry (compare Fig. 1b, f). A delayed decay of YFP-Vpr fluorescence suggests the existence of an additional post-fusion step that triggers dissociation of YFP-Vpr from the viral core.

**Fig. 2:**
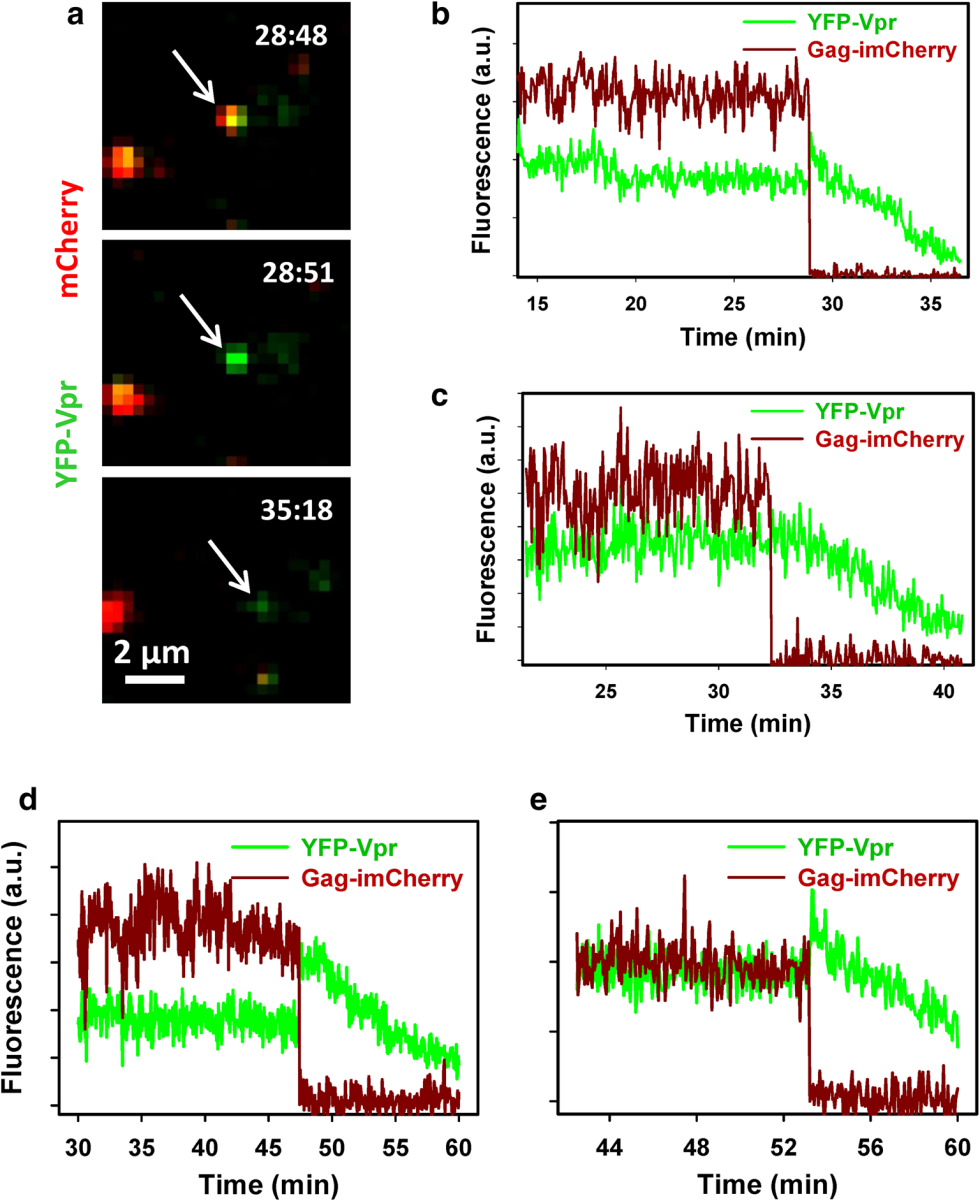
Loss of YFP-Vpr after viral fusion mediated by HXB2 envelope glycoprotein. **a** Snapshots of entry and fusion of an HXB2 Env-pseudotyped particle co-labeled with YFP-Vpr (*green*) and Gag-imCherry (*red*). Viruses were pre-bound to CV-1 cells expressing CD4 and CXCR4 and incubated for 1 h at 37 °C to allow fusion, which was manifested by an abrupt loss of the mCherry marker. Post-fusion decay of the YFP-Vpr signal is evident from the lowest image panel. **b** Single particle tracking of the virus in **a**, showing a virtually instantaneous loss of mCherry (*dark red*) followed by a gradual decay of the YFP signal (*green*). **c**–**e** Examples of HXB2 pp fusion (release of mCherry) with subsequent reduction in the YFP-Vpr fluorescence for pseudoviruses produced using pR8ΔEnv (**c**) and psPAX2 (**d**, **e**) vectors

Single virus tracking demonstrated that a gradual loss of YFP-Vpr signal after viral fusion was universally observed for particles pseudotyped with HXB2 Env glycoprotein (Fig. 2). As observed previously, the pH-independent fusion mediated by HXB2 Env occurred at delayed time-points after initiation of entry, compared to low pH-triggered fusion mediated by VSV-G or ASLV Env ([10, 29–31] and see below). However, in all cases, the formation of the fusion pore was manifested in an abrupt loss of mCherry and transient increase in the YFP-Vpr signal followed by a slow decay (Figs. 1, 2).

**Fig. 3:**
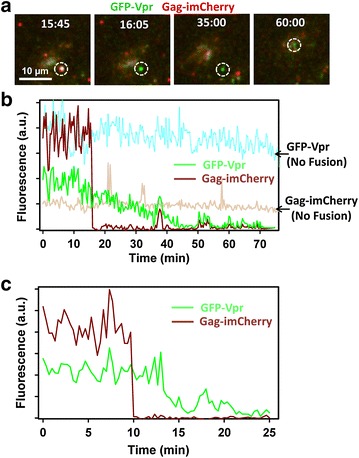
Single VSV-G pseudovirus fusion results in loss of GFP-Vpr from viral core. VSVpp co-labeled with Gag-imCherry (content marker, *red*) and GFP-Vpr (core marker, *green*) were pre-bound to CV-1 cells and allowed to fuse at 37 °C in Fluorobrite DMEM medium under 5 % CO_2_. **a** Images of mCherry release from single virus followed by gradual loss of the GFP-Vpr signal. **b** *Dark red and green* traces show sum fluorescence of mCherry and GFP channels, respectively, obtain by tracking the virus shown in **a**. For comparison, fluorescence intensities of mCherry and GFP for a non-fusing particle are shown (*beige and cyan* traces, respectively). **c** Single virus tracking results of another fusing VSVpp. Occasional spikes in fluorescence (for example, at the 38 min time point) are due to a transient overlap of the particle of interest with either another particle or with cell’s autofluorescent features

It should be noted, however, that reliable quantification of the YFP-Vpr loss from post-fusion particles is confounded by the absence of an additional reference channel, which would enable reliable tracking of post-fusion particles. However, gradual loss of YFP-Vpr from all post-fusion viral cores suggests that, in most cases, this effect reflected Vpr shedding and was not due to particle deviation from a focal plane. To rule out axial displacement as the cause for loss of the Vpr signal, 3D time-lapse images of viruses and cells were acquired. In addition, to better match the conditions previously reported to enable detection of Vpr-labeled particles in the cytoplasm, hours after initiating virus fusion [2, 5, 24], we: (1) used GFP-Vpr and Gag-imCherry labeled viruses pseudotyped with VSV-G (VSVpp); (2) carried out imaging in a complete growth medium equilibrated with 5 % CO_2_ to improve cell viability; (3) visualized viral entry/fusion with a wide-field DeltaVision microscope and acquired multiple Z-stacks to ensure that the entire cell volume was imaged; and (4) deconvolved images prior to single particle tracking. VSVpp fused efficiently with CV-1 cells, as evidenced by abrupt loss of the mCherry signal at different times after initiation of entry/fusion (exemplified in Fig. 3a). As observed for other pseudoviruses using two- or three-dimensional imaging (Figs. 1, 2), the GFP-Vpr signal gradually decayed after the viral content release (Fig. 3a, b). The time required for complete loss of the GFP-Vpr signal varied from a few minutes (Fig. 3c) to 1 h, and this loss of signal was not caused by photobleaching (Fig. 3b, cyan and beige curves). These results rule out axial displacement of post-fusion cores as the reason for vanishing Vpr fluorescence and imply that subtle differences in imaging conditions, such as the imaging buffer and/or CO_2_ level, do not strongly modulate the ability to visualize post-fusion GFP-Vpr puncta.

### YFP-Vpr released from a post-fusion core accumulates in the nucleus

**Fig. 4:**
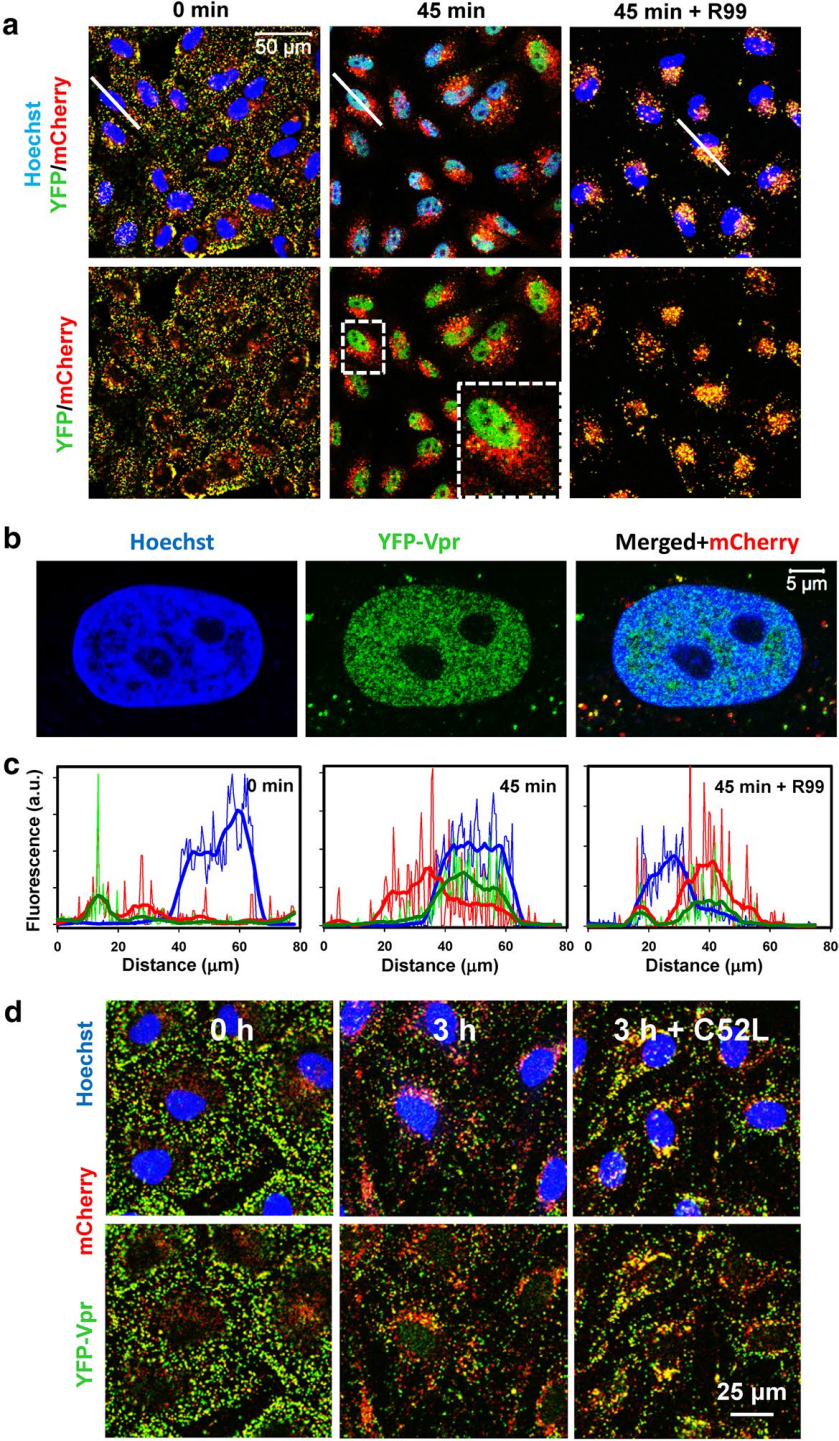
Nuclear accumulation of YFP-Vpr upon co-incubation of viruses with target cells. **a** ASLVpp labeled with YFP-Vpr (*green*) and Gag-imCherry (*red*) were pre-bound to CV-1 cells expressing TVA950 in the cold (*left panels*), and virus entry was initiated by shifting to 37 °C. At 45 min post-initiation, 70 mM of NH_4_Cl was added to block fusion and fully recover the YFP fluorescence in acidic endosomes (*middle panels*). Virus-cell incubation in the presence of fusion inhibitory R99 peptide (50 μg/ml) abrogates nuclear accumulation of YFP-Vpr, but not virus uptake (*right panels*). The *top panels* are three-color (Hoechst/YFP-Vpr/Gag-imCherry) images, while the *bottom panels* show only the YFP-Vpr and Gag-imCherry channels for clarity. The *left* and *middle panels* are the same image field at different time points, while the *right panels* are from a different experiment carried out in the presence of R99. *White lines* are drawn through the nuclei to generate respective intensity profiles shown in **d**. *Inset* in the *lower middle panel* shows the enlarged boxed area. **b** High-resolution confocal image of an optical slice through the middle of the CV-1 cell nucleus stained with Hoechst-33342 after incubation with MOI of ~0.05 of ASLVpp co-labeled with YFP-Vpr and Gag-imCherry. **c** Line histograms through nuclei corresponding to images in (**a**) depict the degree of spatial overlap of YFP-Vpr (*green*), mCherry (*red*) and Hoechst (*blue*) signals before raising the temperature and after 45 min at 37 °C in the absence or in the presence of R99 peptide. **d** Images of YFP-Vpr/Gag-imCherry labeled HXB2 pp particles pre-bound to CV-1-derived target cells before (*left*) and after (*middle*) incubation at 37 °C for 3 h. Parallel samples (*right*) were incubated for 3 h in the presence of 5 μM of HIV-1 fusion inhibitor C52L. Three-color images (*upper panels*) and two-color images (*lower panels*) are shown for the ease of identification of the YFP-Vpr signal within the Hoechst-stained nuclei (*blue*)

Since Vpr has two nuclear localization signals [32], the YFP-Vpr marker released from post-fusion cores is expected to enter the nucleus. Indeed, progressive YFP-Vpr accumulation in the nuclei was observed within 45 min incubation of ASLVpp and cell at 37 °C (Fig. 4a; see also Additional file 1: Movie 1). Spatial redistribution of YFP-Vpr and Gag-imCherry over time is apparent from the linear intensity profiles for lines passing through the nuclear and perinuclear regions (Fig. 4a), as well as from 3D-rendered images (Additional file 2: Movie 2). The relatively scattered green and red puncta at the beginning of the experiment condensed within the nucleoplasm and perinuclear space, respectively, after incubation at 37 °C (Fig. 4c).

The nuclear YFP signal was progressively inhibited by increasing concentrations of the ASLV fusion inhibitor R99 (Additional file 3: Figure S1) or in the presence of NH_4_Cl that raises the endosomal pH (data not shown). Thus, the nuclear YFP-Vpr fluorescence was strictly dependent on the viral fusion activity. The YFP-Vpr was rather evenly distributed within the nucleoplasm and exhibited both diffuse and slightly punctate appearance (Fig. 4b). By contrast, both markers concentrate in the perinuclear region in the presence of R99, which blocks ASLV fusion but not endocytosis (see also Additional file 4: Movie 3).

The post-fusion loss of YFP-Vpr from viral cores and subsequent accumulation in the nucleus were observed for ASLVpp and VSVpp fusing with three different cell lines (Fig. 4; Additional file 3: Figure S2A, B). The nuclear entry of viral YFP-Vpr was observed independent of whether pseudoviruses were produced using a vector that express all HIV-1 proteins except Env (e.g., pR8ΔEnv, Fig. 4) or the Gag-Pol expression vector psPAX2 (e.g. Additional file 3: Figure S2A, B). The apparent shedding of Vpr from post-fusion cores and subsequent nuclear entry were not caused by appendage of bulky YFP marker, since HA-tagged Vpr also accumulated in the nucleus (Additional file 3: Figure S2C). Incubation of double-labeled HXB2 pp with CD4/CXCR4-expressing CV-1 cells also resulted in detectable nuclear YFP-Vpr accumulation, which was inhibited by C52L (Fig. 4d). However, in this case, the nuclear YFP signal was weak and was only observed when a large number of pseudoviruses were attached to target cells. The weak nuclear YFP-Vpr fluorescence is likely due to inefficient HIV-1 fusion. Only ~1 % of double-labeled HXB2 pp released their content marker in single virus imaging experiments, as previously observed [10, 26]. Inefficient nuclear accumulation of YFP-Vpr upon HXB2 pp entry suggests that several fusion events must occur in each cell in order for detectable amounts of this marker to accumulate in the nucleus (see also the fluorescence correlation spectroscopy results below).

Collectively, our results strongly imply that the nuclear YFP-Vpr originates from post-fusion HIV-1 cores and that the loss of this marker after viral fusion is largely independent of the cell type and envelope glycoproteins mediating low pH-dependent (ASLV, VSV) or pH-independent (HXB2) fusion.

### Nuclear Vpr accumulation correlates with the extent of viral fusion

**Fig. 5:**
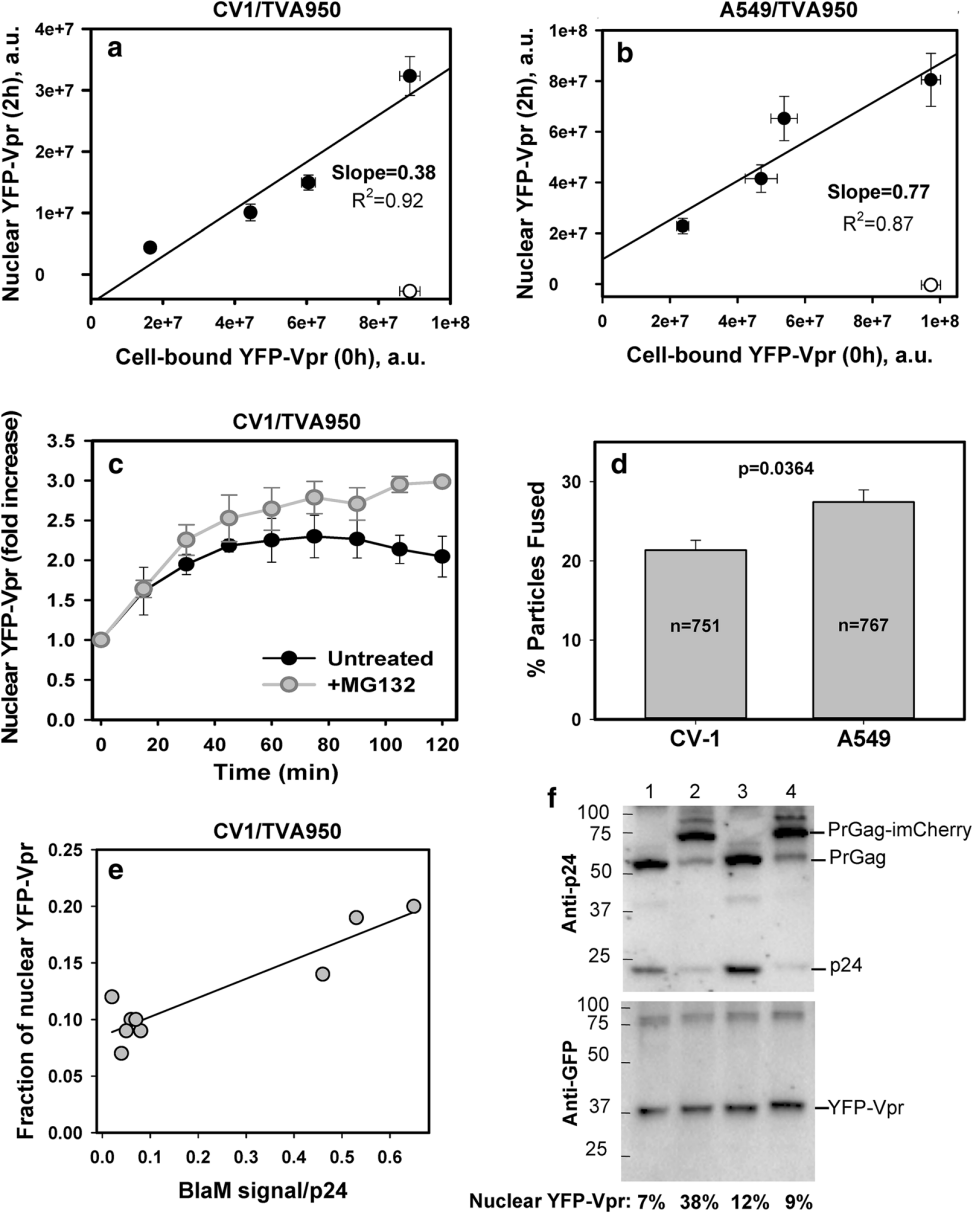
Nuclear accumulation of YFP-Vpr correlates with virus input. **a**, **b** Varying dilutions of YFP-Vpr/Gag-imCherry labeled ASLVpp were pre-bound to CV-1/TVA950 (**a**) or A549/TVA950 (**b**) cells in the cold followed by incubation at 37 °C for 2 h in the presence of 20 μΜ MG132. The nuclear YFP-Vpr was measured in fixed samples in the absence (*filled circles*) or in the presence (*open circles*) of 50 μg/ml R99 peptide. Data are means and SEM from four image fields. Linear regression lines are shown. **c** ASLVpp fusion-mediated nuclear accumulation of YFP-Vpr in CV-1/TVA950 cells in the absence (*filled circles*) or in the presence (*gray circles*) of 20 μΜ MG132. Data represent the ratio of nuclear YFP-Vpr to the signal prior to initiation of fusion. **d** The fusion efficiency of ASLVpp/YFP-Vpr/Gag-imCherry with CV-1/TVA950 or A549/TVA950 cells, as determined by single particle tracking. *Bars* are means and SEM from three experiments each, with total number of dual-labeled viral particles indicated. **e** Correlation between the fraction of viral YFP-Vpr accumulated in the nucleus after 2 h at 37 °C (see the legend to **a**) and the slope of viral fusion (BlaM signal) vs the viral p24 input (see Additional file 3: Figure S6). Every point represents a distinct ASLVpp or VSVpp preparation. **f** ASLVpp (*lanes 1, 2*) and VSVpp (*lanes 3, 4*) were produced using the wild-type HIV-1 R9ΔEnv backbone and co-labeled with YFP-Vpr (and BlaM-Vpr to enable measurements of virus-cell fusion shown in Fig. 5a). The viruses either contained (*odd lanes*) or lacked (*even lanes*) Gag-imCherry. Equal amounts of p24 from virus preparations were subjected to SDS-PAGE and blotted for p24 (HIV IG antibody) or YFP-Vpr (GFP antibody). The loading order: *1* ASLVpp/YFP-Vpr, *2* ASLVpp/YFP-Vpr/Gag-imCherry, *3* VSVpp/YFP-Vpr and *4* VSVpp/YFP-Vpr/Gag-imCherry. The numbers are the respective fractions (%) of viral YFP-Vpr that entered the nucleus in live cell experiments within 2 h

Analysis of the relationship between the nuclear YFP-Vpr and viral fusion showed that the nuclear signal was directly proportional to virus input and, therefore, to the number of fusion events. In two different cell types, the integrated YFP-Vpr signal in the nucleus at the end of a 2 h-incubation at 37 °C correlated with the number of cell-bound YFP-Vpr/Gag-imCherry labeled ASLVpp prior to raising the temperature (plotted as the total cell-associated YFP-Vpr fluorescence in Fig. 5a, b). The slow decay of nuclear Vpr signal observed at longer incubation times could be prevented by the proteasome inhibitor, MG132, which enhanced the nuclear YFP-Vpr signal without affecting the kinetics of its accumulation (Fig. 5c). We found that 38 and 77 % of YFP-Vpr from cell-bound viruses entered the nuclei of CV-1/TVA950 or A549/TVA950 cells, respectively (as reflected by slopes in Fig. 5a, b). If YFP-Vpr is completely lost from post-fusion cores (as suggested by single virus imaging data shown in Figs. 1, 2, 3) and enters the nucleus, these numbers (38 and 77 %) would correspond to the respective fusion efficiencies. As shown in Fig. 5d, visual identification and single particle tracking of co-labeled viruses confirmed the significantly greater fusion efficiency of ASLVpp in A549/TVA950 (27 %) vs. CV-1/TVA950 (21 %) cells. The higher fusion efficiency with A549 cells was consistently observed by both techniques for different viral preparations (Additional file 3: Figure S3), suggesting that the more efficient nuclear delivery of YFP-Vpr in A549/TVA950 cells is, at least partially, due to the higher fusion activity compared to CV-1/TVA950 cells.

The greater apparent extents of fusion estimated from the nuclear YFP-Vpr signals in CV-1 and A549 cells (38 and 77 %, Fig. 5a, b) compared to 21–27 % fusion determined by single particle tracking (Fig. 5d) could be due to: (1) incomplete colocalization of the YFP-Vpr and Gag-imCherry markers in pseudoviruses ([26] and see below), which precludes the detection of all single particle fusion events; (2) shorter incubation time in single virus imaging experiments compared to the YFP-Vpr accumulation experiments; (3) inability to properly track all fusing particles owing to the insufficiently high signal-to-background ratio and/or temporal resolution of live cell imaging experiments; and (4) an inherently more robust signal associated with bulk signal measurements, as in the case of nuclear accumulation estimation. Additionally, even immature HIV-1 particles present in the pseudovirus population (e.g. [26] and see below) can contribute to the nuclear YFP-Vpr signal (see below), whereas the lack of releasable mCherry in immature particles precludes detection of single virus by imaging.

### YFP-Vpr shedding from post-fusion cores occurs independently of co-labeling with Gag-imCherry

It is worth emphasizing that the fraction of virus-associated YFP-Vpr that entered the nuclei was variable and was usually lower than the fractions shown in Fig. 5. This parameter varied between isogenic pseudovirus preparations and could be as low as 9 % (see below). We asked whether virus co-labeling with Gag-imCherry could somehow destabilize the YFP-Vpr-core interactions. Since virus fusion cannot be detected without a releasable content marker, we incorporated the β-lactamase-Vpr (BlaM-Vpr) chimera into pseudoviruses labeled with YFP-Vpr and measured their fusion activity using a BlaM assay [10, 16, 33]. This strategy enabled the comparison of the nuclear YFP-Vpr accumulation (Additional file 3: Figure S4) and the fusion activity (measured by the BlaM assay) for the same viral preparation.

Similar to YFP-Vpr/Gag-imCherry labeled ASLVpp and VSVpp (Fig. 5; Additional file 3: Figure S3), particles containing BlaM-Vpr and YFP-Vpr, or containing only GFP-Vpr, delivered variable fractions (between 9 and 40 %) of the fluorescent marker into the nucleus after 2 h at 37 °C (Additional file 3: Figures S5 and S6). The overlapping ranges of nuclear entry efficiency for Vpr-only labeled and YFP-Vpr/Gag-imCherry labeled viruses indicate that the latter marker may not considerably promote shedding of fluorescent Vpr. In agreement with single particle imaging data (Fig. 3), substitution of YFP-Vpr with GFP-Vpr did not affect the extent of nuclear accumulation (Additional file 3: Figure S6C and E). Overall, the fraction of YFP-Vpr entering the nucleus tended to correlate with the fusion efficiency, as determined by a BlaM assay (Fig. 5e and see below). This result is consistent with the notion that the extent of viral fusion, but not the subsequent YFP-Vpr shedding, limits the nuclear delivery of this marker. We must stress, however, that it is difficult to evaluate the relative contributions of the fusion efficiency and the extent of YFP-Vpr shedding from cores to the fraction of Vpr recovered in the nucleus.

To assess whether different extents of viral YFP-Vpr delivery into the nucleus were due to the variable incorporation of this marker into viral preparations, ASLVpp and VSVpp labeled with YFP-Vpr or YFP-Vpr/Gag-imCherry were analyzed by SDS-PAGE. Blotting for GFP showed that YFP-Vpr incorporation was fairly uniform across four preparations (Fig. 5f), implying that variable YFP-Vpr content did not contribute to the efficiency of its nuclear delivery (Fig. 5f, numbers on the bottom). Moreover, since the nuclear YFP-Vpr signal is normalized to the total viral YFP-Vpr input (e.g., Fig. 5a, b), variability in YFP-Vpr content among different virus preparations is not a confounding factor. These results further support the notion that the extent of nuclear YFP-Vpr delivery is determined, at least in part, by the efficiency of viral fusion.

Notably, co-incorporation of YFP-Vpr and Gag-imCherry impaired virus maturation, as evidenced by the reduced intensity of the p24 and increased intensity of the Pr55 and PrGag-imCherry bands in double-labeled compared to single labeled samples (Fig. 5f, compare lanes 1 and 3 with lanes 2 and 4). The less efficient maturation of viral particles containing both YFP-Vpr and Gag-imCherry is consistent with a considerable fraction of glass-immobilized particles retaining the Gag-imCherry marker in the presence of Triton X-100 (TX-100) ([26] and see data below). Considering that immature particles pseudotyped with VSV-G, and likely with ASLV Env, may still be fusogenic [33], we asked whether these particles can contribute to the nuclear accumulation of YFP-Vpr. To address this possibility, immature VSVpp labeled with YFP-Vpr were produced in the presence of the HIV-1 protease inhibitor, saquinavir, which blocked the cleavage of Gag polyprotein (Additional file 3: Figure S7). Incubation of immature pseudoviruses with CV-1- and A549-derived cells resulted in nuclear entry of ~4 % of the total viral YFP-Vpr (Additional file 3: Figure S7). This observation confirms the ability of immature VSV-G pseudotyped particles to undergo fusion and shows that fusing immature particles can, in principle, contribute to the nuclear delivery of YFP-Vpr. It is thus possible that the differences in the extent of virus maturation in different preparations contribute to the variability of nuclear YFP-Vpr fractions observed in our experiments.

### Fluorescently labeled Vpr is not released from lysed virions

**Fig. 6:**
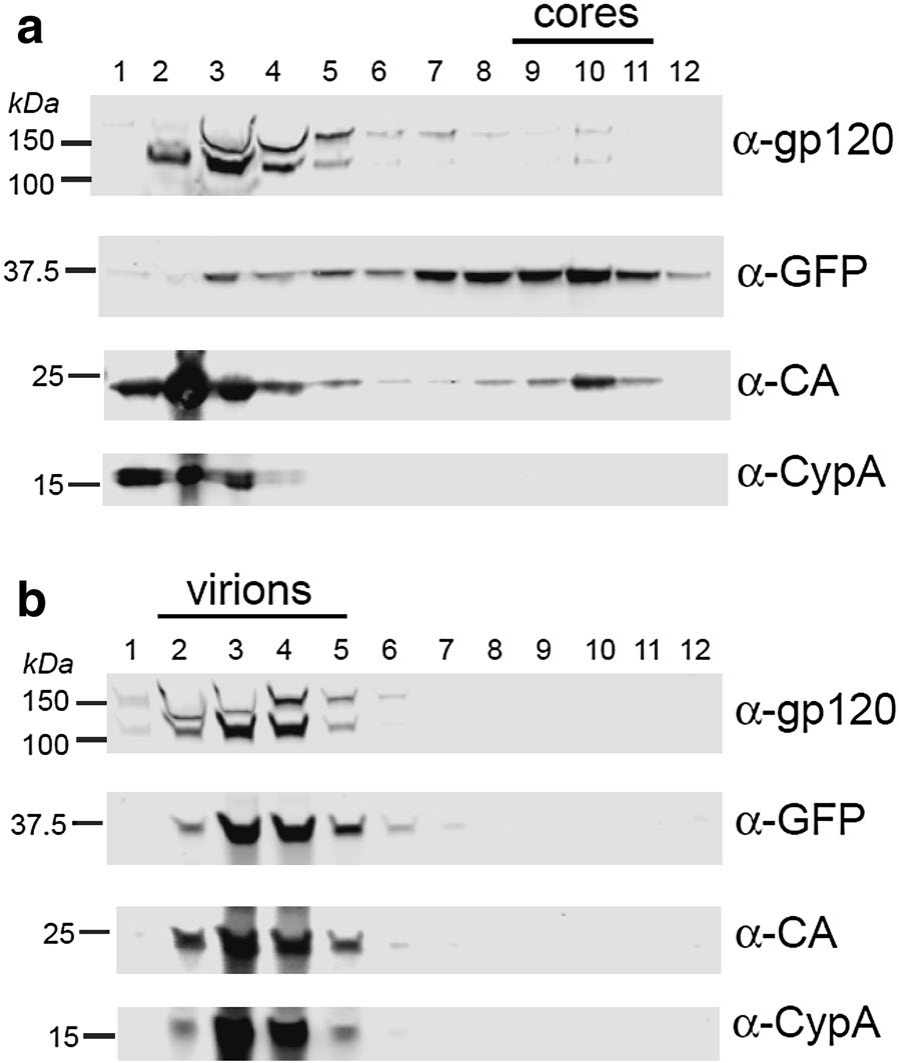
Retention of GFP-Vpr by purified HIV-1 cores. **a** Analyses of HIV-1 cores released upon centrifugation of concentrated HIV-1 particles through a layer of Triton X-100 detergent. Proteins in the fractions were concentrated by TCA precipitation and subjected to SDS-PAGE and immunoblotted for gp120, GFP, HIV-1 CA, and cyclophilin A (CypA). Numbered fractions were collected from the top to the bottom of the gradient. Viral cores sedimented to fractions 9–11. **b** Analysis of GFP-Vpr-labeled HIV-1 particles in parallel following centrifugation on a gradient lacking the detergent layer. Intact HIV-1 particles sedimented to fractions 2–5

Considering the striking loss of fluorescently labeled Vpr from post-fusion cores, we asked if GFP-Vpr is similarly dissociated from HIV-1 cores released by TX-100 treatment and equilibrium sedimentation on a sucrose gradient [34]. Fractions were analyzed by immunoblotting for HIV-1 gp120, CA, GFP-Vpr (GFP-specific antibody) and cyclophilin A (CypA, Fig. 6a). While a large quantity of CA was detected at the top fractions of the gradient, a small fraction of the total CA was present in fractions 9–11, corresponding to the density of intact HIV-1 cores, as is normally observed [35]. GFP-Vpr co-sedimented with the cores (fractions 9–11), whereas CypA was primarily found at the top of the gradient. In control experiments, intact viral particles sedimented with fractions 2–5 and contained all four viral markers (Fig. 6b).

**Fig. 7:**
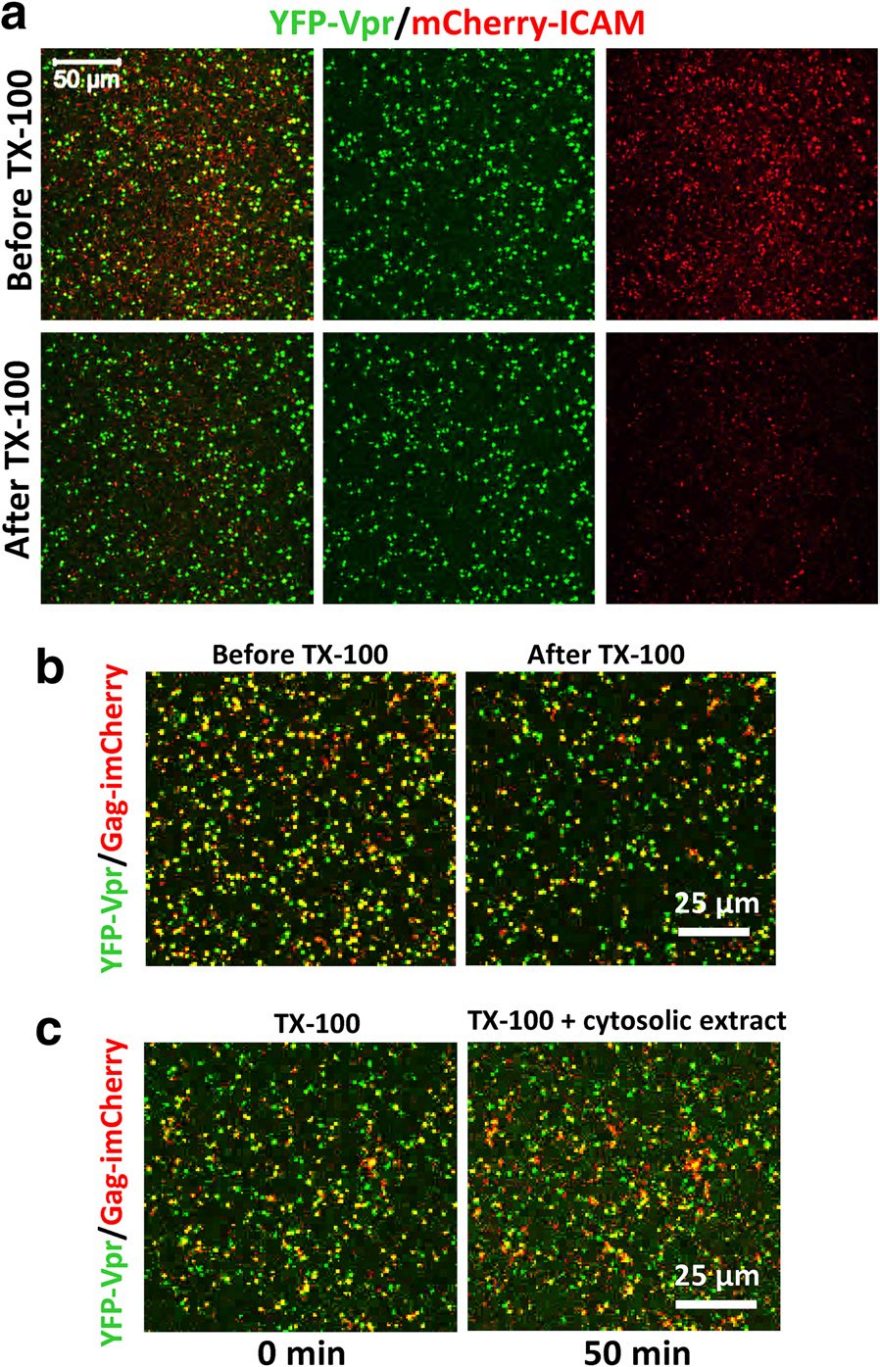
YFP-Vpr is not shed from immobilized viruses after detergent lysis and exposure to cytosolic extract. **a** Removal of the viral membrane with TX-100. VSVpp co-labeled with YFP-Vpr (*green*, core) and mCherry-ICAM (*red*, membrane) were diluted in PBS and bound to Cell-Tak™ coated chamber coverslips (Lab-Tek) for 30 min at 4 °C. Images were acquired before and after the addition of 0.1 % TX-100 for 1 min, which effectively removed the viral membrane, as seen by the loss of the mCherry-ICAM signal. **b** TX-100 treatment (0.1 %) of immobilized ASLVpp co-labeled with YFP-Vpr and Gag-imCherry resulted in loss of mCherry from a fraction (35 %) of particles that properly matured. **c** Naked cores remaining after TX-100 lysis of immobilized ASLVpp were washed with PBS and incubated with 0.5 mg of cytosolic extract from rhesus monkey liver cells diluted in 0.3 ml PBS for 50 min at 37 °C. In all panels, the same image fields are shown before and after TX-100 or cytosol treatment

To further test the strength of YFP-Vpr association with the HIV-1 core in vitro, fluorescently labeled pseudoviruses attached to a Cell-Tak™ coated coverslip were lysed by TX-100 for 1 min. This treatment removed the viral membrane, as evidenced by the loss of the membrane marker from particles co-labeled with YFP-Vpr and mCherry fused to the transmembrane domain of ICAM-1 (mCherry-ICAM, Fig. 7a). However, YFP-Vpr was not released from these viruses. This result is consistent with partial retention of GFP-Vpr in equilibrium sedimentation experiments (Fig. 6a). Perhaps a combination of a longer detergent exposure and centrifugal force used in the latter experiments destabilizes the cores and promotes both CA and GFP-Vpr shedding.

YFP-Vpr was not released from lysed particles co-labeled with the viral content marker Gag-imCherry, which was lost immediately upon exposure to detergent (Fig. 7b). Interestingly, the YFP-Vpr signal of membrane-stripped immobilized viruses remained virtually unchanged after incubation with cytosolic extract from rhesus monkey or human cells for 50 min at 37 °C (Fig. 7c and data not shown, respectively). The lack of YFP-Vpr release from lysed pseudoviruses is in stark contrast with the relatively quick shedding of this marker in live cell imaging experiments and its subsequent accumulation in the nucleus.

### YFP-Vpr released from post-fusion cores quickly enters the nucleus

**Fig. 8:**
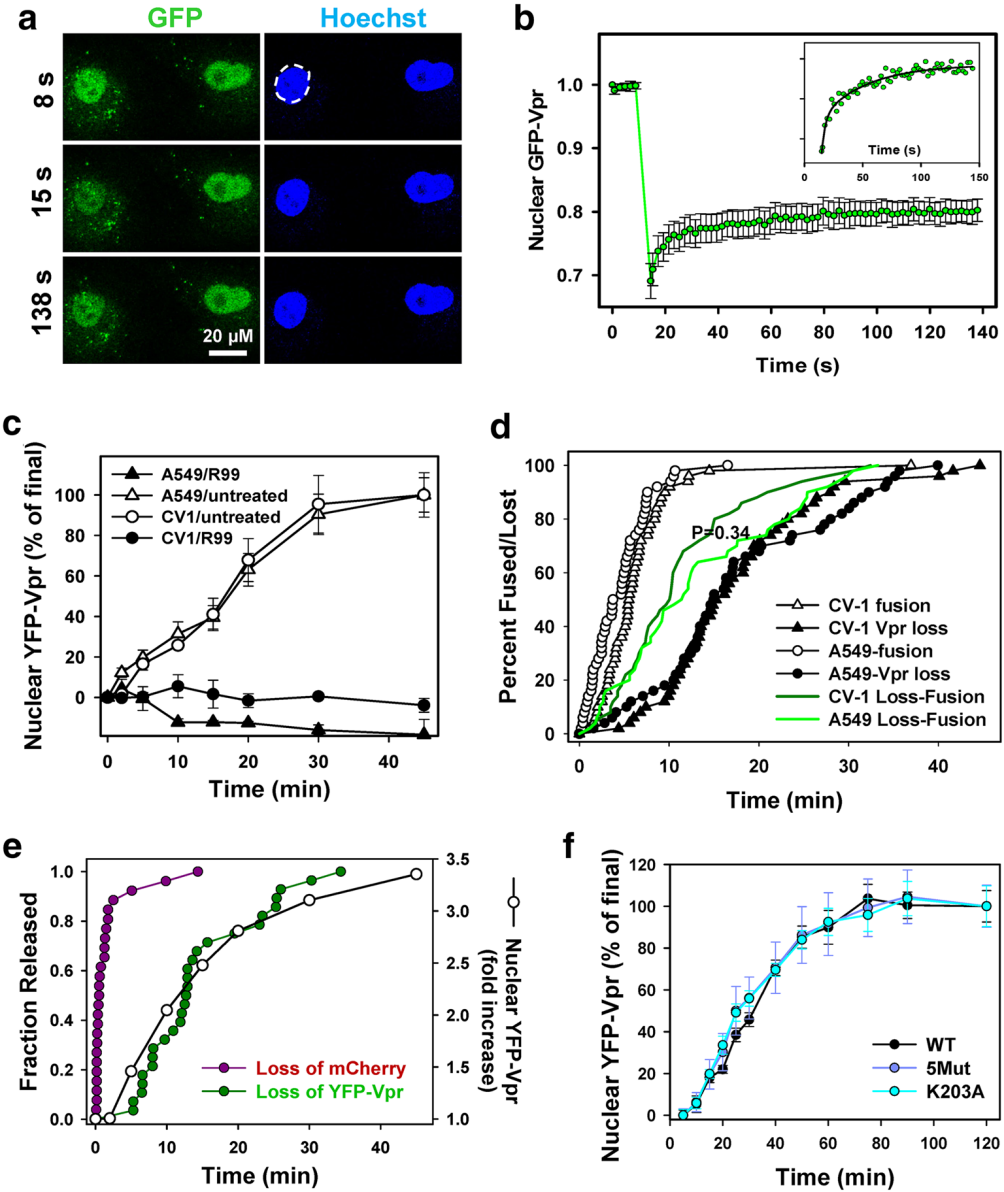
YFP-Vpr shedding is rate-limiting for nuclear entry and is not modulated by capsid stability. **a** Nuclear GFP-Vpr and Hoechst fluorescence before and after GFP photobleaching. **b** Fluorescence recovery after photobleaching. Circles are normalized means and SEM of 10 nuclei. *Inset* The GFP-Vpr signal recovery after photobleaching (*circles*), the line is a double-exponential fit to the data. **c** Kinetics of Vpr nuclear accumulation in CV-1/TVA950 and A549/TVA950 cells in the presence or absence of 50 μg/ml of R99 peptide. **d** Kinetics of single ASLVpp fusion and YFP-Vpr shedding in CV-1- or A549-derived cells measured as the time-point of mCherry disappearance from dual-labeled ASLVpp and complete loss of YFP-Vpr, respectively. *Circles* represent normalized cumulative plots for signal disappearance from ASLVpp. Lifetimes of post-fusion cores were measured as the difference in disappearance times of mCherry and YFP signals for the same particle. **e** Synchronized fusion of ASLV from endosomes. ASLVpp was allowed to enter CV-1-derived cells for 45 min at 37 °C in the presence of 70 mM NH_4_Cl. Viral fusion was initiated by replacing NH_4_Cl with imaging buffer, and the kinetics of fusion (release of mCherry) and loss of YFP-Vpr was measured (*left axis*). The corresponding appearance of YFP-Vpr in the nucleus in the same imaging field was determined as a fold-increase over that prior to initiation of synchronous fusion from endosomes (*open circles*, *right axis*). **f** CV-1/TVA950 cells inoculated with YFP-Vpr-labeled VSVpp containing either the wild-type (WT) HIV-1 capsid (R9 backbone) or one of the two capsid mutants, K203A (destabilizing) and 5Mut (stabilizing). The amount of cell-bound viruses was equalized based on cell-associated YFP-Vpr fluorescence and viruses were allowed to fuse for 2 h at 37 °C in the presence of 20 μΜ MG132. The nuclear YFP-Vpr signal was measured at indicated time intervals and normalized to the value at 2 h. Data-points are means and SEM from four image fields each

To determine whether YFP-Vpr released from fused virions shuttles between the cytoplasm and the nucleus, we performed fluorescence recovery after photobleaching (FRAP) experiments. Cells were incubated with GFP-Vpr labeled ASLVpp at 37 °C (2 h) to allow viral fusion and GFP-Vpr entry into the nucleus (Fig. 8a). Regions of interest corresponding to the entire nuclei, as determined by Hoechst staining (dashed circle), were partially photobleached by a brief exposure to the maximal power of a 488 nm laser. The marginal recovery of the nuclear GFP-Vpr signal after photobleaching (Fig. 8b) shows that the majority of this protein accumulates in the nucleus within 2 h post-infection. The kinetics of fluorescence recovery was bi-exponential, with characteristic times of 2.6 ± 0.3 and 84 ± 34 s (n = 10), as determined by curve fitting. The faster diffusion component was not reliably resolved because its characteristic time was close to the time required to photobleach a significant portion of nuclear GFP-Vpr. FRAP measurements thus demonstrate the ability of the cytosolic GFP-Vpr to quickly enter the nucleus and suggest that shedding of this marker from individual post-fusion cores occurring over the course of several minutes to 1 h (Figs. 1, 2, 3), may be rate-limiting.

The time course of YFP-Vpr accumulation in the nucleus was independent of the cell type. Identical rates of nuclear fluorescence increase were detected in the simian CV-1- and human A549-derived cell lines (Fig. 8c). The invariant longevity of post-fusion YFP-Vpr signal in cell lines of different origin (Fig. 8c, d, P = 0.34) implies that cytosolic factors/processes that appear to promote shedding of this marker are not host-specific. Single particle tracking data also showed that ASLVpp fusion (release of mCherry) and subsequent loss of YFP-Vpr occurred with the nearly identical kinetics in these two cell lines (Fig. 8d). Consistent with the notion that Vpr is quickly delivered to the nucleus, the half-times for the loss of single YFP-Vpr puncta after fusion (Fig. 8d, filled symbols) and for the appearance of diffuse nuclear fluorescence (Fig. 8c) were surprisingly close: 15.2 and 16.2 min, respectively. We also evaluated the characteristic lifetime of single post-fusion YFP-Vpr puncta by plotting the lag time between the release of mCherry and loss of YFP-Vpr. The post-fusion loss of YFP-Vpr in CV-1/TVA950 and A549/TVA950 cells occurred with half-times of 10.0 and 11.4 min, respectively (Fig. 8d, solid lines). Thus, post-fusion loss of YFP-Vpr appears rate-limiting for the nuclear import of this marker which occurs with a half-time of ≤80 s (Fig. 8b).

Given the limited imaging time, we cannot completely rule out the possibility that post-fusion Vpr puncta may remain detectable for longer than 1 h. For example, a rare particle shown in Additional file 3: Figure S8A lost approximately 70 % of the initial GFP-Vpr signal shortly after fusion, but the remaining signal appeared steady for ~37 min and could potentially be detected at later times. In order to better estimate the longevity of Vpr puncta after fusion, we extended the imaging time to 2 h. In addition, 3D time-lapse images were acquired and deconvolved to improve the signal-to-background ratio for single GFP-Vpr puncta (Fig. 3; Additional file 3: Figure S8B). However, in spite of the improved ability to visualize faint particles compared to 2D imaging (analyzed in Fig. 8d), reliable tracking was limited to under 1 h after initiation of fusion for the vast majority of cytosolic cores (Additional file 3: Figure S8B, solid line). Under these conditions, individual post-fusion cores were observed to shed Vpr with a half time of ~20 min.

To more accurately assess the kinetics of nuclear entry of YFP-Vpr after viral fusion with endosomes, we synchronized ASLVpp fusion by taking advantage of the ASLV’s ability to survive the NH_4_Cl block and undergo quick fusion upon removal of NH_4_Cl [27, 36]. Double-labeled ASLVpp were allowed to enter CV-1-derived cells for 45 min in the presence of NH_4_Cl, and viral fusion was initiated by replacing the NH_4_Cl-containing buffer with a regular buffer. Removal of NH_4_Cl results in uniform acidification of all intracellular compartments and thereby synchronously triggers ASLVpp fusion [27]. Single virus imaging confirmed rapid fusion with nearly 90 % of fusion (mCherry release) occurring within the first 150 s after NH_4_Cl removal (Fig. 8e, magenta circles). Subsequent accumulation of YFP-Vpr in the nucleus occurred with a half-time of 6.3 min, and this curve overlapped with the kinetics of the YFP-Vpr loss from individual particles after the synchronized fusion (Fig. 8e, open vs. green circles). In other words, YFP-Vpr appears to enter the nucleus shortly after dissociating from the post-fusion cores.

### YFP-Vpr shedding does not correlate with HIV-1 capsid stability

Next, we asked whether the stability of the HIV-1 capsid could modulate its ability to retain YFP-Vpr after fusion, as measured by the nuclear YFP signal. Toward this goal, VSVpp containing the wild-type HIV-1 R9 backbone (WT) and two capsid mutants, K203A and 5Mut, were produced and tested. The K203A mutation markedly destabilizes mature capsids [34], whereas the 5Mut capsid containing five substitutions is more stable compared to WT capsid in an in vitro assay [37]. In our infection system, K203A and 5Mut were approximately 4 orders and 1 order of magnitude, respectively, less infectious than WT. To avoid potential effects of Gag-imCherry incorporation on Vpr shedding, pseudoviruses were labeled only with YFP-Vpr. Equal amounts of labeled WT and mutant viruses, as evidenced by the nearly identical total cell-associated YFP fluorescence signals (1.1 × 10^8^ arbitrary intensity units), were pre-bound to cells in the cold. Virus entry/fusion was triggered by raising the temperature, and the nuclear YFP-Vpr signal was measured at indicated time points. We reasoned that, since the nuclear import of Vpr is not rate-limiting (Fig. 8e), the rate of its accumulation in the nucleus should reflect the rate of YFP-Vpr shedding from the post-fusion cores. Of note, the kinetics of viral fusion, which could also contribute to the time course of nuclear entry of YFP-Vpr, was not affected by point mutations in the viral capsid (data not shown). The normalized kinetics of nuclear YFP-Vpr accumulation for WT and the two mutants were superimposable (Fig. 8f), suggesting that the YFP-Vpr shedding from post-fusion cores is not affected by the capsid stability. We also did not detect consistent effects of reverse transcriptase inhibitors, nevirapine and azidothymidine, on the ASLVpp-mediated nuclear accumulation of YFP-Vpr in CV-1- and A549-derived cells (Additional file 3: Figure S9).

### Fluorescently labeled Vpr associates with large cellular complexes

**Fig. 9:**
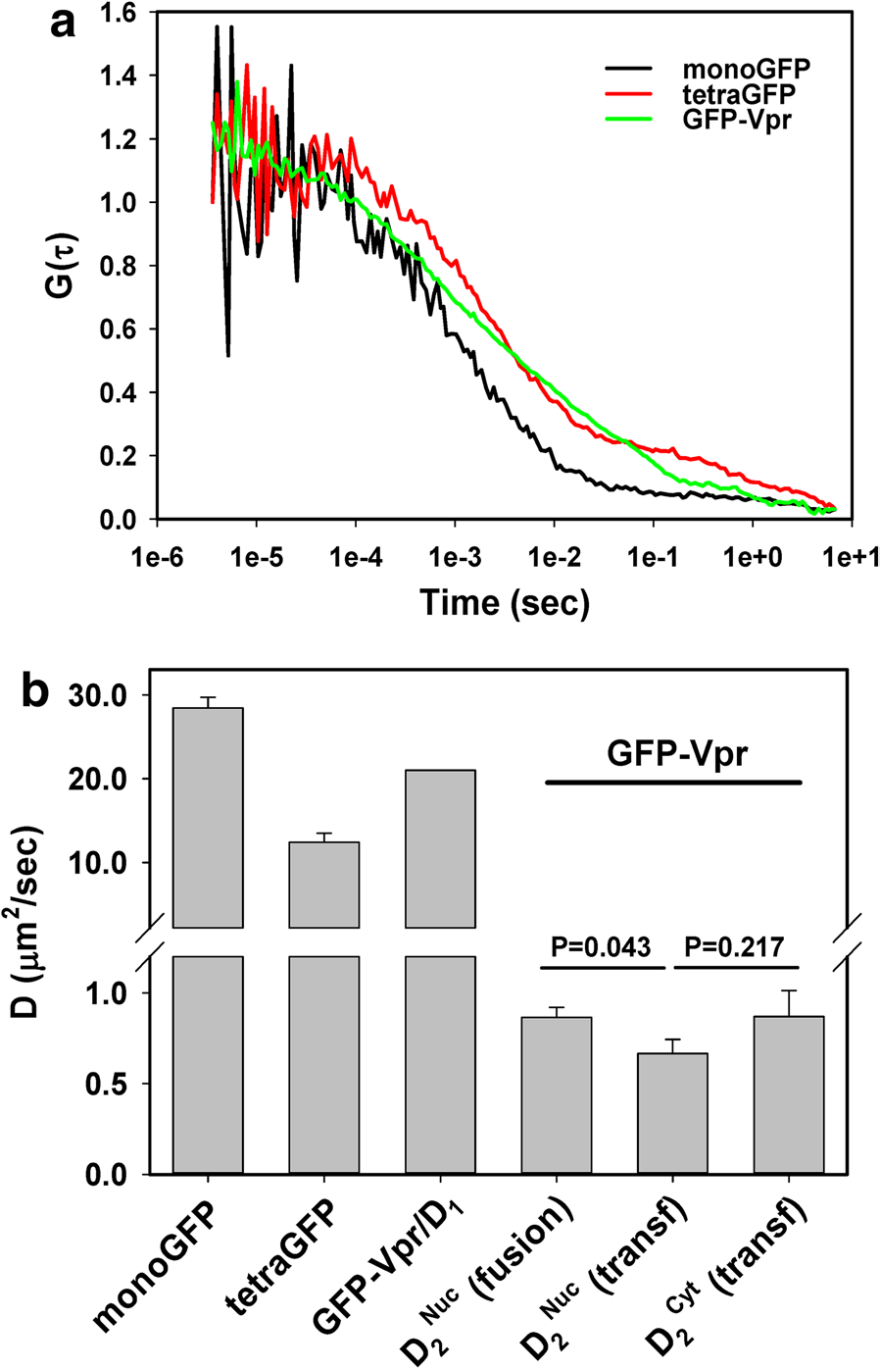
Cellular GFP-Vpr mobility analysis by fluorescence correlation spectroscopy. **a** Representative autocorrelation curves for monomeric and tetrameric GFP expressed in A549 cells, as well as for nuclear GFP-Vpr delivered through fusion of ASLVpp co-labeled with GFP-Vpr and Gag-imCherry. **b** Diffusion coefficients obtained by curve fitting the autocorrelation plots in **a**, as described in “Methods” and in Additional file 3: Figure S10. Monomeric and tetrameric GFP curves were fit with a 3D single-component diffusion model, whereas the GFP-Vpr curves could only be fit with a 2-component diffusion equation. The faster diffusion coefficient D_1_ was assumed to correspond to an GFP-Vpr monomer and was fixed for curve fitting purposes in order to obtain D_2_ coefficient. Similar analysis was performed for GFP-Vpr expressed in A549 cells by transient transfection. Data are means and SEM from 6 to 12 experiments. Possible reasons for the unexpectedly large difference in D for a monomer and a tetramer compared to the predicted D − 1/(MW)^1/3^ relationship are discussed in [39]

To examine the mobility pattern of nuclear Vpr, we employed fluorescence correlation spectroscopy (FCS). To match the excitation and emission wavelengths to those used for calibrating the confocal volume (see “Methods”; Additional file 3: Figure S10), we used GFP-Vpr instead of YFP-Vpr. First, monomeric or tetrameric variants of GFP described in [38] were expressed in A549/TVA950 cells by transient transfection. The diffusion coefficients (D) of monomeric and tetrameric species at different subcellular locations, including the nucleus, were determined from the experimentally obtained autocorrelation curves (Fig. 9a; Additional file 3: Figure S10). Both monomeric and tetrameric GFP moved as single species with the diffusion coefficients of 28 ± 1 and 12 ± 1 μm^2^/s, respectively. The obtained D values are consistent with those reported in the literature [39].

We next performed FCS measurements in the A549/TVA950 cell nuclei after incubation with GFP-Vpr/Gag-imCherry labeled ASLVpp at 37 °C to allow fusion and nuclear delivery of GFP-Vpr. The shape of autocorrelation curves obtained from multiple locations within the nuclei revealed the existence of more than one diffusing GFP-Vpr species (Fig. 9a). The results were well fitted using a two diffusing species model. To simplify data analysis, we assumed that the faster diffusion coefficient (D_1_) corresponds to an GFP-Vpr monomer, free diffusion of which should occur at a rate slightly slower than for GFP due to the size difference (41 vs. 27 kDa [19]). Assuming that the diffusion time is roughly proportional to cubic root of the molecular mass, D_1_ = 21 μm^2^/s was used as the diffusion coefficient of GFP-Vpr. Curve fitting performed using this fixed D_1_ value yielded a markedly slower diffusion coefficient for the second species, D_2_: 0.87 ± 0.06 μm^2^/s.

Our results demonstrate the existence of two major forms of GFP-Vpr in A549/TVA950 cells—a monomer and a very large oligomeric complex. Considering that D is inversely proportional to MW^1/3^, the ~24-fold difference between D_1_ and D_2_ translates into complexes that are 14,000-fold larger than the 4 × 10^3^ Da GFP-Vpr monomer, i.e. 6 × 10^8^ Da. To rule out the unlikely possibility that large nuclear complexes represent GFP-Vpr-labeled PICs, we ectopically expressed GFP-Vpr in the absence of other viral proteins in A549 cells. FCS measurements revealed the same two-species diffusion pattern with similar D_2_ values in the nucleus and the cytoplasm (Fig. 9b). This finding suggests that large Vpr-containing complexes are present in both cytoplasm and nucleoplasm.

FCS analysis also yields the number of fluorescent molecules in a confocal volume. With the viral inputs corresponding to the medium/high range in Fig. 5B, the estimated nuclear GFP-Vpr concentration in the confocal volume of ~0.8 × 10^−15^ liter was 18 nM. Based on high resolution confocal imaging of A549 cells, we calculated the average nuclear volume to be ~1000 μm^3^. Thus, the total number of GFP-Vpr molecules per nucleus should be ~10,000. Assuming that each virus carries about 300 molecules of fluorescent GFP-Vpr, more than 30 fusion events per cell that culminate in a complete loss of this marker from post-fusion cores would have to occur to deliver ~10^4^ GFP-Vpr into the nucleus. This result is consistent with the relatively low sensitivity of the nuclear Vpr accumulation assay.

## Discussion

Tracking of single YFP-Vpr-labeled particle revealed that this marker dissociates from the post-fusion HIV-1 cores and, consistent with previous reports [32, 40, 41], enters into the cell nucleus. The YFP-Vpr shedding and its concomitant accumulation in the nucleus were reproducibly observed in target cells of different origin and using different virus labeling schemes or viral fusion glycoproteins. We found no evidence for the effect of capsid stability on the kinetics of nuclear YFP-Vpr accumulation, indicating that this marker is shed from post-fusion cores prior to capsid uncoating. However, a lag between the loss of mCherry and onset of YFP-Vpr decay exhibited by a fraction of post-fusion cores (Fig. 1) indicates that an additional post-fusion event may be needed to release this marker. In contrast to short-lived post-fusion YFP-Vpr puncta, detergent-permeabilized viral particles attached to a coverslip did not lose YFP-Vpr in the presence or absence of the cytosolic extract from rhesus monkey or human liver cells. Retention of labeled Vpr by permeabilized viruses implies that shedding of this protein from post-fusion cores is induced by active processes occurring in the cytoplasm.

The relatively quick shedding of YFP-Vpr after fusion is in agreement with the previously observed loss of GFP-Vpr puncta within 90 min of HeLa cell infection [42]. However, these results appear discordant with the visualization of GFP-Vpr-labeled post-fusion cores over the course of several hours (e.g. [2, 5]) and with biochemical evidence that Vpr is associated with the HIV-1 PICs [43–47]. Our biochemical data confirmed that a minor fraction of GFP-Vpr was recovered with purified capsids while the majority of CA and GFP-Vpr was in soluble fractions (Fig. 6a). It is thus possible that a small pool of labeled Vpr, which is below the limit of detection by fluorescence microscopy, is tightly associated with the viral core. It remains to be established whether core-associated or shed Vpr molecules play a role in HIV-1 infection.

The loss of detectable Vpr signal seems at odds with other studies that were able to visualize fluorescent Vpr puncta several hours post infection. Possible explanations include: (1) long lived Vpr puncta are, for the most part, unfused, endosome-resident viruses; (2) only a very small fraction of YFP-Vpr-labeled viral cores survive after fusion, a problem that can be circumvented by using high doses of virus in the fixed cell experiments; however, such a regime is incompatible with real-time single particle tracking; (3) fixed cell imaging offers a better signal-to-background ratio compared to live cell imaging.

Whereas reports of Vpr shedding from HIV-1 cores are scarce, ultrastructural, biochemical and functional studies found that HIV-2- and SIV-encoded Vpx is loosely associated with the core and is readily released after virus entry [48–50] (see [51] for the opposite conclusion). Interestingly, virus-incorporated Vpx is rapidly released from post-fusion cores, accumulates in the nucleus and initiates SAMHD1 degradation in HeLa-derived cells and primary macrophages within a few hours after inoculation [49, 50]. It is thus tempting to speculate that nuclear accumulation of HIV-1 Vpr observed in our study may also play a yet unknown role in establishment of productive infection.

The finding that all tracked particles lost YFP-Vpr within a few minutes after fusion and that the resulting nuclear YFP-Vpr signal correlates with the number of cell-bound viruses suggests that nuclear fluorescence reflects the extent of viral fusion. This assay is robust, but is not sensitive, since several fusion events must occur to produce a detectable level of nuclear Vpr. Perhaps as a result of this, the nuclear Vpr signal was hardly detectable after fusion of particles pseudotyped with HXB2 Env. In spite of the relatively low sensitivity of this assay, the appearance of nuclear Vpr enables quick assessment of the extent and kinetics of VSVpp and ASLVpp fusion. The determinants for the varied nuclear accumulation efficiency of YFP-Vpr from similar/isogenic viral preparations are currently unknown.

Our FCS measurements of GFP-Vpr mobility in the nucleoplasm imply that this protein associates with giant (~6 × 10^8^ Da) complexes in the cytoplasm and the nucleus. Note, however, that the cytoplasmic and nuclear Vpr complexes could be distinct. Vpr is known to bind to large protein complexes consisting of DCAF1 (55 kDa), Cullin4A (88 kDa), DDB1 (130 kDa), UNG2 (35 kDa) and SLX4 (~200 kDa) [52–54], as well as to DNA, RNA and chromatin [55–58]. The FCS results are consistent with our FRAP measurements, which also support the existence of two fluorescent Vpr species with vastly different diffusion coefficients. It is conceivable that large cytosolic Vpr complexes enter the nucleus much slower than Vpr monomers. Our findings are in general agreement with the previous work [59] reporting large Vpr-GFP homo-oligomers (1000× larger than a monomer). In the light of Vpr homo-oligomerization, further studies are needed to define the nature of slowly diffusing species of GFP-Vpr observed in our FCS experiments. It should also be kept in mind that a GFP-tag can alter the sub-cellular distribution of Vpr, apparently by modulating Vpr-host protein interactions. Indeed, the distribution and function of unlabeled Vpr and Vpr-GFP appear distinct from those of GFP-Vpr [47, 59, 60] (but see [61] for similar distributions of WT and fluorescent Vpr).

HIV-1 Vpr exerts multiple effects on cells, including cell cycle arrest [54, 62, 63]. Vpr expression is essential for optimal HIV-1 replication in macrophages, but not in CD4^+^ T cells or cell lines [64–69]. Based on its strong karyophilic properties [32, 40, 70] and retention in PICs [43–47], Vpr has been implicated in early steps of infection, including the nuclear entry of viral DNA/PICs and regulation of viral gene expression [40, 41, 47, 62, 63, 70–75]. As a rule, however, these effects were observed upon over-expression or exogenous addition of Vpr to productively infected cells (e.g. [54, 66, 76, 77]). It is not clear whether small amounts of HIV-associated Vpr released into the cytoplasm can modulate the virus’ ability to establish productive infection. The ability of virus-derived Vpr to induce NF-κB and AP-1 signaling [78, 79], inhibit antiviral responses [54, 80] and induce G_2_ cell cycle arrest in the absence of productive infection [75, 81] has been documented. It is therefore conceivable that quick dissociation of Vpr from incoming HIV-1 cores and nuclear entry helps dampen innate antiviral responses and/or optimizes post-fusion steps of HIV-1 entry. Further studies are needed to investigate this possibility.

## Conclusions

Single virus tracking, confocal imaging and fluorescence correlation spectroscopy reveal that fluorescently-tagged Vpr is shed from virtually all post-fusion HIV-1 cores and gets transported to the nucleus where it forms large complexes with host proteins. Synchronized ASLV Env-mediated fusion shows that the nuclear import of virus-derived YFP-Vpr is rapid. While the YFP-Vpr loss is delayed relative to the release of a viral content marker, its signal usually vanishes within a few minutes after fusion, indicating that the Vpr loss precedes capsid uncoating, which is thought to occur on a much slower time scale. Since the efficiency of nuclear Vpr delivery correlates with the extent of viral fusion, the nuclear signal from fluorescent Vpr can be used to estimate the viral fusion activity. Further studies are needed to delineate the determinants of Vpr retention by the viral cores and a possible role of shed Vpr in post-fusion steps of HIV-1 entry.

## Methods

### Cell lines, plasmids and reagents

Human alveolar adenocarcinoma A549, human embryonic kidney HEK293T/17, and African green monkey kidney CV-1 cell lines were obtained from the ATCC (Manassas, VA, USA). HeLa-derived TZM-bl cells expressing CD4, CXCR4 and CCR5 (donated by Drs. J.C. Kappes and X. Wu [82]) were obtained from the NIH AIDS Research and Reference Reagent Program. CV-1/TVA950, TZM-bl/TVA and CV-1/CD4/CXCR4 cells have been described previously [26, 27]. A549/TVA950 cells were obtained by transducing A549 cells with VSV-G pseudotyped retroviral vectors pCMMP-TVA950, as described previously [36]. Cells expressing high levels of TVA receptor were obtained by staining with the subgroup A ASLV SU-IgG fusion protein [36] and a goat anti-rabbit FITC-conjugated antibody (Sigma, St. Louis, MO, USA) and sorting using a FACS Aria II (BD Biosciences, San Jose, CA, USA) flow cytometer. Sorted cells were grown in high glucose Dulbecco’s Modified Eagle Medium (DMEM, Mediatech, Manassas, VA, USA) with 10 % Fetal Bovine Serum (FBS, Sigma, St. Louis, MO, USA) and 100 U/ml penicillin–streptomycin (Gemini Bio-Products, Sacramento, CA, USA). For HEK 293T/17 cells the propagation medium was supplemented with 0.5 mg/ml G418 sulfate (Mediatech).

The expression vectors pR8ΔEnv, pMM310 (encoding for BlaM-Vpr), pcRev, HIV-1 Gag-imCherryΔEnv and pMDG (encoding for VSV G) were described previously [26, 83]. The YFP-Vpr and GFP-Vpr plasmids were a gift from Dr. T. Hope (Northwestern University). The pR9ΔEnv vectors expressing the capsid mutants K203A and 5Mut have been described previously [34, 37]. The psPAX2 lentiviral packaging vector was obtained from the NIH AIDS Research and Reference Reagent Program (Cat. #11348); pLKO.1-puro-shScr (scrambled) lentiviral vector was from Sigma, and pLVX-mKate2 lentiviral vector was a gift from Dr. A. Brass (University of Massachusetts). To obtain psPAX2-Gag-imCherry plasmid, standard overlap extension PCR methods were used to insert a polylinker containing *Mlu*I and *Not*I restriction sites between MA and CA domains of the Gag. The fluorescent protein mCherry was PCR amplified and inserted into the polylinker region. The resulting clone contains an 8-amino-acid SQNYPIVQ protease catalytic site flanking the mCherry sequence.

Calf skin collagen, Bafilomycin A1 and rabbit polyclonal anti-HA antibody were purchased from Sigma-Aldrich. Cy5-labeled goat anti-rabbit IgG was from KPL (Gaithersburg, MD, USA). Hoechst-33342, Live Cell Imaging Buffer, Fluorobrite DMEM media, CCF4-AM substrate, and rhesus monkey liver cytosol (catalog number RHCY-PL) were from Life Technologies (Grand Island, NY, USA). MG132 was from Calbiochem (Billerica, MA, USA) and Cell-Tak™ cell and tissue adhesive was purchased from BD Biosciences (San Jose, CA, USA). The R99 peptide (~95 % purity by HPLC) derived from ASLV-A glycoprotein envelope [84]. HIV-IG (Catalog #3957, donated by Dr. L. Barbosa) and rabbit HIV-1 Vpr (1-50) antiserum (Cat#11836, donated by Dr. J. Kopp) polyclonal antibodies were obtained from NIH AIDS Research and Reference Reagent Program. Mouse anti-GFP and mouse anti-beta-lactamase antibodies were purchased from Clontech (Mountain View, CA, USA) and QED Bioscience (San Diego, CA, USA), respectively. AlphaLISA immunoassay kit was from PerkinElmer (Waltham, MA, USA). The HIV-1 gp41 glycoprotein-derived C52L recombinant peptide was a kind gift from Dr. Min Lu (University of New Jersey).

### Pseudovirus production, labeling and characterization

Pseudovirus production and titration were described previously [26]. Briefly, fluorescently labeled pseudoviruses were produced by transfecting HEK293T/17 cells with 1 μg of pR8ΔEnv or pR9ΔEnv, 2 μg HIV-1-Gag-imCherryΔEnv or psPAX2-Gag-imCherry, 2 μg of YFP-Vpr or GFP-Vpr, and 2 μg of glycoprotein envelope expression vector using JetPrime Transfection reagent (VWR, Radnor, PA, USA). psPAX2-based pseudoviruses were produced by transfecting the HEK293T/17 cells with 1.5 μg psPAX2, 1.5 μg psPAX2-Gag-imCherry, 2 μg of YFP-Vpr, 2 μg of pLKO.1-puro-shScr or pLVX-mKate2, and 3 μg of the viral envelope glycoprotein expression vector. To incorporate beta-lactamase-Vpr (BlaM-Vpr) chimera into fluorescent viruses, 1 μg of YFP-Vpr and 1 μg of BlaM-Vpr were co-transfected along with other plasmids. The virus-containing medium was collected at 48 h post-transfection, passed through a 0.45 µm filter, aliquoted, frozen and stored at −80 °C. The infectious titer was determined by a β-Gal assay (for details, see [10]) in TZM-bl/TVA cells.

### p24 measurements and Western blotting

The viruses were concentrated using Lenti-X concentrator (Clontech), resuspended in PBS and lysed with 0.5 % Triton X-100 for 30 min at room temperature. The HIV-1 p24 quantity was determined either by ELISA, as described previously [85] or using an AlphaLISA immunoassay kit. Equal amounts of p24 were loaded onto 10 % polyacrylamide gel (Bio-Rad, Hercules, CA, USA). Proteins were transferred onto a nitrocellulose membrane, blocked with 10 % Blotting-grade Blocker (Bio-Rad) for 1 h at room temperature and probed with the indicated antibodies. Precision Plus Protein Standards (Kaleidoscope™ Bio-Rad) were used as molecular weight markers.

### Isolation and analysis of HIV-1 cores

Viral cores were isolated from concentrated HIV-1 particles by the spin-through procedure on 20–70 % sucrose gradients [86]. Proteins in gradient fractions were concentrated by TCA precipitation, resuspended in Laemmli buffer, and subjected to SDS-PAGE and immunoblotting using the following antibodies: mouse anti-gp120 (902, from NIH AIDS Research and Reference Reagent Program, Cat. #522), rabbit anti-cyclophilin A (Millipore, Cat. #07-313), mouse anti-GFP (MP Biomedicals, Cat. #7302-1) and rabbit anti-CA (produced by immunization with purified recombinant HIV-1 CA). After probing with the appropriate IR dye-conjugated secondary antibodies, bands were detected by scanning the blots with a LI-COR Odyssey instrument. Bio-rad “All Blue Standards” were used as molecular weight markers.

### Vpr immunostaining

ASLVpp produced with HA-tagged Vpr were spun onto CV-1/TVA950 cells in the cold and entry was initiated by the addition of live cell imaging buffer with 2 % FBS at 37 °C. At the end of 1 h post-entry, cells were washed with PBS at room temperature, fixed with 2 % PFA for 20 min, permeabilized with 0.5 % Triton X-100 for 15 min, blocked with 10 % FBS for 30 min, and incubated overnight at 4 °C with 12 µg/ml of anti-HA polyclonal antibody (Sigma-Aldrich). Secondary immunostaining was performed by incubating cells with 5 µg/ml of Cy5-labeled goat anti-rabbit IgG (KPL, Gaithersburg, MD, USA) for 1 h at room temperature. Nuclei were stained with 4 µg/ml Hoechst-33342 during the secondary staining step.

### β-Lactamase (BlaM) viral fusion assay

The pseudovirus fusion with target cells was measured using the BlaM assay, as described previously [10, 83]. Briefly, cells cultured in 96-well black clear-bottom plates were pre-treated for 30 min, at 37 °C, 5 % CO_2_ with growth medium containing 20 μM MG132. The viruses were bound to target cells by centrifugation at 4 °C for 30 min at 1550×*g*, in the absence of MG132. After the virus binding step, cells were washed once with cold PBS and incubated in live cell imaging buffer/2 % FBS/20 μM MG132 at 37 °C for 90 min. The fusion reaction was stopped by placing the plates on ice, and the media was replaced with the BlaM substrate, CCF4-AM (Invitrogen). Cells were incubated at 11 °C overnight, and the BlaM activity was determined from the ratio of coumarin (blue) and fluorescein (green) fluorescence signals, using a SpectraMaxi3 fluorescence plate reader (Molecular Devices, Sunnyvale, CA, USA).

### Imaging of viral entry, Vpr nuclear accumulation and image analysis

Single particle viral fusion experiments were performed on cells grown on collagen-coated glass-bottom Petri dishes (MatTek, MA, USA) in FluoroBrite DMEM with 10 % FBS to 90 % confluency. Viral post-fusion YFP-Vpr nuclear accumulation titrations were performed on cells grown on collagen-coated 96-well black glass-bottom sensoplates from Greiner Bio-One (Monroe, NC, USA), and pre-treated for 30 min before virus addition with 20 µM MG132 added to the growth media. In either case the cells were chilled on ice and washed with cold PBS. Pseudoviruses diluted to a desired MOI (from 0.05 to 1.0 for CV-1-derived cells) were bound to cells by spinoculation at 1500×*g*, 4 °C for 20 min. The cells were washed twice with cold PBS, and virus entry was initiated by adding pre-warmed live cell imaging buffer containing 2 % FBS for single particle fusion experiments and, additionally, 20 µM MG132 for YFP-Vpr nuclear accumulation titrations. Virus entry was allowed to proceed for 45 min to 1 h for single particle fusion experiments, and for 2 h for titration experiments at 37 °C, where entry was terminated by placing the cells on ice. Cells samples for titrations were then fixed with 2 % paraformaldehyde in PBS for 20 min at room temperature. To measure the extent of virus binding, parallel samples with matched MOIs were fixed on ice for 10 min with cold 2 % paraformaldehyde solution immediately after virus spinoculation followed by an additional 20 min incubation at room temperature with the fixative. All samples were washed with PBS after fixation, and imaged in PBS containing 70 mM NH_4_Cl in order to raise endosomal pH and fully recover the quenched YFP-Vpr signal in low pH endosomes. For live cell experiments, nuclei were stained for 30 min with 10 μg/ml Hoechst-33342 prior to virus binding; for fixed cell experiments, the dye was added after the fixation step.

Images were acquired on a Zeiss LSM780 confocal microscope using a C-Apo 40×/1.2NA water-immersion objective. One to three z-stacks were imaged for live cell experiments, and fixed cells were imaged with multiple Z-stacks, 0.5 μm apart. Hoechst-33342, GFP, YFP and mCherry were excited at 405, 488, 514 and 594 nm, respectively. In the YFP-Vpr nuclear accumulation titrations four to five different fields were imaged for each condition. In order to eliminate emission bleed-through from the Hoechst signal into the GFP or YFP channel, images were collected using two separate tracks in the line-switching mode on Zen imaging software package (Carl Zeiss). The DefiniteFocus™ module (Carl Zeiss) was utilized to maintain focus for all time-course imaging, which was performed within a thermally controlled chamber at 37 °C on the microscope stage.

Quantification of nuclear delivery of Vpr was performed as follows. The total number of cell-bound viral particles per field at t = 0 was determined by intensity- and size-based thresholding in 3D using the object-finder routine in Volocity (Perkin Elmer, Waltham, MA, USA). Nuclear accumulation of Vpr was determined by measuring GFP- or YFP-Vpr signal in the total nuclear volume defined by Hoechst-33342 staining. The fidelity of this procedure was also established for samples imaged in a live cell setting with fewer Z-stacks (~2 μm spacing) to reduce total imaging time, with no variation in data quality. The extent of Vpr accumulation was then calculated by generating correlation plots between total GFP- or YFP-Vpr signals from cell-bound particles *vs* total post-fusion nuclear Vpr signal imaged in matched acquisition configurations. All single viral fusion events were identified by visual inspection, and single particle-tracking was performed using Volocity.

### Wide-field deconvolution imaging of virus fusion

CV-1 cells were grown in Fluorobrite DMEM medium and seeded on collagen-coated MatTek glass-bottom dishes, as described above. VSVpp co-labeled with GFP-Vpr and Gag-imCherry were diluted in cold Fluorobrite DMEM supplemented with 10 % FBS and spun onto cells, as described above. The cells were washed, mounted onto a microscope stage, and virus entry was initiated by the addition of pre-warmed Fluorobrite/10 % FBS and imaged at 37 °C under 5 % CO_2_. Images were collected every 20 s with a UPlanFLN 40× Oil/1.3 NA objective on a personal DeltaVision microscope equipped with an EM-CCD camera (Photometrics). Typically, 12–13 Z-stacks (spaced by 1 µm) were acquired per time-point. Axial drift of samples was compensated with the Ultimate Focus module. The images were deconvolved, using SoftWorx software, and viral particles were tracked in 3D with Volocity, as described above.

### Synchronized ASLVpp fusion with endosomes

ASLVpp were pre-bound to CV-1/TVA950 cells in the cold, as described above, and virus endocytosis was initiated by adding warm imaging buffer with 70 mM NH_4_Cl to block fusion. After 45 min at 37 °C, viral fusion was triggered by replacing NH_4_Cl with imaging buffer using a stage-mounted local perfusion system [27], and the resulting synchronized viral fusion was imaged for ~15 min at 37 °C. To measure the rate of nuclear accumulation of YFP-Vpr following the synchronized viral fusion, the buffer exchange was performed outside the microscope chamber, after which the cells were rapidly reintroduced onto the stage and imaged every 2 min at 37 °C.

### Lysis of immobilized viruses in vitro

Pseudoviruses were diluted in PBS and allowed to bind to poly-l-lysine coated coverslips (Nunc Lab-Tek, Thermo Scientific) for 30 min at 4 °C. The coverslips were washed with PBS, and viruses were imaged before and after lysis with 0.1 % TX-100 for 1 min at room temperature. The coverslips were then washed with PBS four times and incubated with 1.67 mg/ml of rhesus liver cytosolic extract (Life Technologies, Grand Island, NY, USA) supplemented with 100 µM ATP and 1 mM DTT in PBS. Samples were imaged for 50 min at 37 °C.

### Fluorescence recovery after photobleaching (FRAP)

FRAP was utilized to determine the rate of nuclear import of cytosolic GFP-Vpr delivered from labeled pseudoviruses. ASLVpp fusion was allowed to proceed for 2 h, as time sufficient to reach quasi-equilibrium between the cytosolic and nuclear Vpr pools. Entire nuclear regions of selected cells were photobleached for 7 s by the 488 nm laser beam at maximum power within a nuclear volume demarcated by Hoechst staining, and fluorescence recovery was recorded using a low intensity beam, essentially as described in [38]. Fluorescence recovery curves were fit with a double-exponential model.

### Fluorescence correlation spectroscopy

Fluorescence correlation spectroscopy (FCS) measurements were performed with the Zen FCS module on a Zeiss LSM780 microscope, essentially as described previously [38]. Briefly, we used a designated C-Apo 40×/1.2NA water-immersion objective. The confocal volume was determined using a series of Atto-488 dilutions. In control experiments, A549/TVA950 cells grown on glass-bottom Petri dishes were transfected with plasmid expressing the monomeric or tetrameric GFP proteins described in [38]. Alternatively, the cells were allowed to fuse with GFP-Vpr/Gag-imCherry labeled ASLVpp for 60 min at 37 °C, as described above, and the mobility of GFP-Vpr in the cell nuclei was analyzed by FCS at room temperature to minimize the cell movement artifacts. The 488 nm laser beam (attenuated by ~100-fold) was focused on selected positions within the nucleus or the cytoplasm, and fluorescence signals were acquired from each location in five sessions each lasting 10 s. The average autocorrelation curves for each location were fit with a normal 3D diffusion model using the QuickFit 3.0 software package available from http://www.dkfz.de/Macromol/quickfit/. Autocorrelation curves for monomeric and tetrameric GFP constructs expressed in the cytoplasm and nuclei were fitted using a single component 3D diffusion model with a triplet state. Data for GFP-Vpr were analyzed assuming two diffusing species.

### Statistical analysis

The degree of statistical significance was determined by the Mann–Whitney Rank test and p < 0.05 was considered significant.

## Additional files

10.1186/s12977-015-0215-z Nuclear accumulation of YFP-Vpr shed from post-fusion cytosolic HIV-1 cores. ASLVpp bearing the HIV-1 core label YFP-Vpr (green) and the content marker Gag-imCherry (red) were bound to CV-1/TVA950 cells in the cold. The cells were brought to a temperature-controlled microscope stage at 37 °C and entry was initiated by the addition of warm buffer. Images were collected every 4 s. Viral fusion, followed by nuclear accumulation of YFP-Vpr shed from post-fusion cytosolic cores, proceeded to completion in ~ 45 min (see also Figs. 3A and 7C and 7D in the main text).
10.1186/s12977-015-0215-z A 3D view of a CV-1/TVA950 cell incubated with ASLV pseudoviruses co-labeled with YFP-Vpr (green) and Gag-imCherry (red) under fusion-permissive conditions. The cell nucleus is labeled with Hoechst-33342 (blue, shown intermittently). Nuclear YFP-Vpr signal is apparent.
10.1186/s12977-015-0215-z Supplemental results (Figures S1 to S10) showing the nuclear accumulation of labeled Vpr under different conditions, including reverse transcriptase inhibitors, as well as the kinetic of loss of GFP-Vpr from single particles following image deconvolution and calibration of FCS setup.
10.1186/s12977-015-0215-z A 3D view of a CV-1/TVA950 cell incubated with ASLV pseudoviruses co-labeled with YFP-Vpr (green) and Gag-imCherry (red) in the presence of the ASLV fusion inhibitor R99. The cell nucleus is labeled with Hoechst-33342 (blue, shown intermittently). Nuclear YFP-Vpr signal is not detected.

## Abbreviations

ASLV: avian sarcoma and leukosis virus
BlaM: beta-lactamase
FCS: fluorescence correlation spectroscopy
FRAP: fluorescence recovery after photobleaching
PIC: pre-integration complex
Vpr: viral protein R
VSV: vesicular stomatitis virus
WT: wild type
