# Supplementary material for: Fluorescent protein-tagged Vpr dissociates from HIV-1 core after viral fusion and rapidly enters the cell nucleus
Source: Retrovirology. 2015 Oct 29;12:88. doi: 10.1186/s12977-015-0215-z (PMC4625717; doi:10.1186/s12977-015-0215-z)
Supplement: Supplementary file 3 — 10.1186/s12977-015-0215-z Supplemental results (Figures S1 to S10) showing the nuclear accumulation of labeled Vpr under different conditions, including reverse transcriptase inhibitors, as well as the kinetic of loss of GFP-Vpr from single particles following image deconvolution and calibration of FCS setup. [file 12977_2015_215_MOESM3_ESM.pdf]

Additional File 1 Movie 1. Nuclear accumulation of YFP-Vpr shed from post-fusion cytosolic HIV-1 cores. ASLVpp bearing the HIV-1 core label YFP-Vpr (green) and the content marker Gag-imCherry (red) were bound to CV-1/TVA950 cells in the cold. The cells were brought to a temperature-controlled microscope stage at 37°C and entry was initiated by the addition of warm buffer. Images were collected every 4 sec. Viral fusion, followed by nuclear accumulation of YFP-Vpr shed from post-fusion cytosolic cores, proceeded to completion in ~45 min (see also Figs. 3A and 7C and 7D in the main text).

Additional File 2 Movie 2. Nuclear accumulation of YFP-Vpr. A 3D view of a CV-1/TVA950 cell incubated with ASLV pseudoviruses co-labeled with YFP-Vpr (green) and Gag-imCherry (red) under fusion-permissive conditions. The cell nucleus is labeled with Hoechst-33342 (blue, shown intermittently). Nuclear YFP-Vpr signal is apparent.

Additional File 3 Supplemental Figures 1 to 10. Supplemental results showing the nuclear accumulation of labeled Vpr under different conditions, including reverse transcriptase inhibitors, as well as the kinetic of loss of GFP-Vpr from single particles following image deconvolution and calibration of FCS setup.

Figure S1. Dose-response plots for R99 inhibition of ASLVpp-mediated nuclear entry of YFP-Vpr in CV-1-derived cells. Ratios of YFP-Vpr signal in the nucleus at 45 min to that at initiation of viral entry (fold-increase) are plotted on Y-axis. Data are mean and SEM from five image fields each.

Figure S2. Nuclear accumulation of Vpr as a result of viral fusion with different cell types. (A, B) Nuclear Vpr delivered *via* fusion of VSVpp (based on the psPAX2 vector) co-labeled with YFP-Vpr (green) and Gag-imCherry (red) into TVA950-expressing TZM-bl (A) and A549 (B) cells. Cell nuclei were stained with Hoechst-33342 (blue). Samples were fixed at 2 h post viral entry, and images were collected on a Zeiss LSM780 confocal microscope using a Plan-Apochromat 63x/1.40NA oil objective at 1 airy unit pinhole aperture. Images represent a single z-slice through the center of the field. (C) Vpr delivery into the nucleus through fusion of unlabeled ASLVpp containing HA-tagged Vpr with CV-1/TVA950 cells. Immunostaining was performed as described in Methods. Left and middle panels, respectively, are cells stained prior to and after ASLVpp/cell co-culture for 1 h. Right panels indicate a sample where fusion was blocked by 50 μg/ml of R99 peptide. Top and bottom rows are the same image field with and without Hoechst-33342 nuclear staining shown for clarity. Images were collected using a Plan-Apochromat 63x/1.40NA oil objective at 1 airy unit pinhole aperture. Images represent a single z-slice through the center of the field.

Figure S3. YFP-Vpr nuclear accumulation after ASLVpp fusion with CV-1/TVA950 and A549/TVA950 cells. Viral fusion and YFP-Vpr nuclear accumulation as a function of virus input were measured in TVA950 expressing CV-1 (A) and A549 (B) cells, as described in Methods. Pseudoviruses were produced by transfecting 293T cells with the pR8ΔEnv expression vector, YFP-Vpr plasmid and psPAX2 vector encoding Gag-imCherry. Filled and open circles are the mean and SEM of four image fields each without and with the fusion inhibitory R99 peptide, respectively. Linear regression lines are shown.

Figure S4. Fusion of pseudoviruses containing YFP-Vpr, BlaM-Vpr and Gag-imCherry leads to nuclear accumulation of YFP-Vpr. ASLVpp used in Fig. 5A and D in the main text were spun onto CV-1/TVA950 cells in the cold and allowed to fuse for 50 min at 37°C.

Figure S5. Fusion of VSVpp containing different HIV-1 backbones leads to nuclear accumulation of GFP-Vpr. GFP-Vpr-labeled VSVpp were produced by transfection of 1×10^{6} 293T cells (100 mm dish) with 15 μg pR9ΔEnv plasmid, 5 μg GFP-Vpr plasmid and 5 μg of the VSV-G plasmid, using the calcium-phosphate protocol. Viruses were spun onto CV-1/TVA950 cells in the cold, and fixed either immediately or at the end of a 2 h-incubation at 37°C in imaging buffer supplemented with 20 μM MG132. (A, B) Images and nuclear GFP-Vpr staining analysis of cells after fusion of VSVpp produced using the HIV-1 pR9ΔEnv vector. Images are single slices through the middle of cells with pre-bound viruses before incubation at 37°C (top row) and post-fusion (bottom row). (C, D) Titrations of VSVpp fusion and nuclear GFP-Vpr accumulation from viruses produced using NL4-3 (C) and pR7ΔEnv (D) backbones. Filled and open circles represent nuclear signals after virus entry in the absence or presence of 100 nM Bafilomycin A1. Data are mean and SEM from four image fields per condition. Linear regression lines are shown.

Figure S6. Extent of Vpr accumulation in the nucleus correlates with viral fusion. VSVpp were produced using different ratios of HIV-1 pR9ΔEnv vector and plasmids expressing YFP-Vpr and BlaM-Vpr (or GFP-Vpr and BlaM-Vpr). Inclusion of BlaM-Vpr allowed the measurements of the efficiency of viral fusion for different preparations (B, D, and F). (A, B) VSVpp produced using 2:0.5:0.5 ratio of pR9ΔEnv, YFP-Vpr and BlaM-Vpr plasmids. The dependences of the nuclear YFP-Vpr signal and the BlaM signal on the viral input are shown in panels A and B, respectively. (C, D) Same as in A and B, but using 3:0.5:0.5 ratio of pR9ΔEnv,

YFP-Vpr and BlaM-Vpr plasmids. (E, F) Same as in C and D, but using GFP-Vpr instead of YFP-Vpr. Open circles represent the BlaM activity or nuclear YFP-Vpr signal in the presence of endosome acidification inhibitors, NH_4Cl or 100 nM Bafilomycin A1 (BafA1), respectively, to block viral fusion. BlaM data are means and SEM from one experiment done in triplicate, and nuclear accumulation data are means and SEM from four image fields per condition. Linear regression lines are shown.

Figure S7. Incubation of cells with immature VSVpp results in reduced nuclear accumulation of YFP-Vpr. Immature pseudoviruses co-labeled with YFP-Vpr and Gag-imCherry were produced in the presence of 80 nM saquinavir (SQV). Viruses were pre-bound to CV-1/TVA50 or A549/TVA950 cells in the cold and incubated for 45 min at 37°C. The dependence of the nuclear YFP-Vpr signal on the virus input is shown for CV-1/TVA950 (A) and A549/TVA950 (B) cells. Control samples (open circles) were incubated in the presence of 100 nM Bafilomycin A1. Data are means and SEM from four image fields per condition. (C) VSVpp co-labeled with Gag-imCherry and YFP-Vpr were produced in the absence or presence of 80 nM SQV, concentrated using LentiX concentrator (Clontech), resuspended in PBS and lysed with 0.5% Triton X-100 for 30 min at room temperature. The HIV-1 p24 content of these preparations was determined by ELISA, and equal amounts of p24 were subjected to SDS-PAGE and blotted for p24 using HIV IG antibody.

Figure S8. Longevity of GFP-Vpr puncta after VSVpp fusion with CV-1 cells. (A) A rare VSVpp fusion event after which the GFP-Vpr signal dropped, but remained detectable throughout the experiment. Images were acquired using a DeltaVision wide-field microscopy system. (B) Kinetics of VSVpp fusion and GFP-Vpr loss. Fusion of VSVpp co-labeled with GFP-Vpr and Gag-imCherry with CV-1 cells was imaged in 3D with a DeltaVision system using

multiple Z-stacks, and images were deconvolved, as described in Methods (see also Fig. 3). The times to release of mCherry (Fusion) and to complete loss of GFP-Vpr (Loss) were measured and plotted as cumulative distributions. The pairwise difference between the time of fusion and loss of GFP-Vpr (Loss – Fusion) for each particle was determined as a measure of longevity of post-fusion GFP-Vpr marker (solid line).

Figure S9. Inhibition of reverse transcription does not affect nuclear accumulation of YFP-Vpr. CV-1- and A549-derived cells were left untreated or were pre-treated with nevirapine (5 μM) or azidothymidine (AZT, 1 μM), which are non-nucleoside and nucleoside analog reverse transcriptase inhibitors, respectively, for 2 h at 37°C. ASLVpp co-labeled with YFP-Vpr and Gag-imCherry were then bound to the cells in the cold, and the viruses were allowed to fuse with cells for 45 min at 37°C in the presence of the respective inhibitors. NH_4Cl at 70 mM was added at the end of incubation to arrest subsequent fusion and dequench YFP signal. Images were then collected to assess nuclear YFP-Vpr accumulation, which was estimated as the fold increase over nuclear signal in corresponding samples with matched viral input prior to fusion. The bars represent means and SEM of five different image fields for each condition.

Figure S10. FCS measurements and calibration. (A-C) Selected autocorrelation curves for A549 cell-expressed monomeric GFP (A), tetrameric GFP (B) and ASLVpp-delivered GFP-Vpr in the nucleus (C). Red lines/crosses show original data, and blue and green lines are fits without and with a non-fluorescent (triplet state) component, using QuickFit3 (residuals are shown in separate panels beneath each graph). (D) Distribution of diffusion coefficients for monomeric and tetrameric GFP in the cytoplasm of A549 cells obtained by curve fitting data exemplified in panels A-C. (E) Calibration of confocal volume for FCS measurements performed using a series of Atto-488 solutions at concentrations indicated on the graph.

<u>Additional File 4 Movie 3</u>. Nuclear accumulation of YFP-Vpr. A 3D view of a CV-1/TVA950 cell incubated with ASLV pseudoviruses co-labeled with YFP-Vpr (green) and Gag-imCherry (red) in the presence of the ASLV fusion inhibitor R99. The cell nucleus is labeled with Hoechst-33342 (blue, shown intermittently). Nuclear YFP-Vpr signal is not detected.

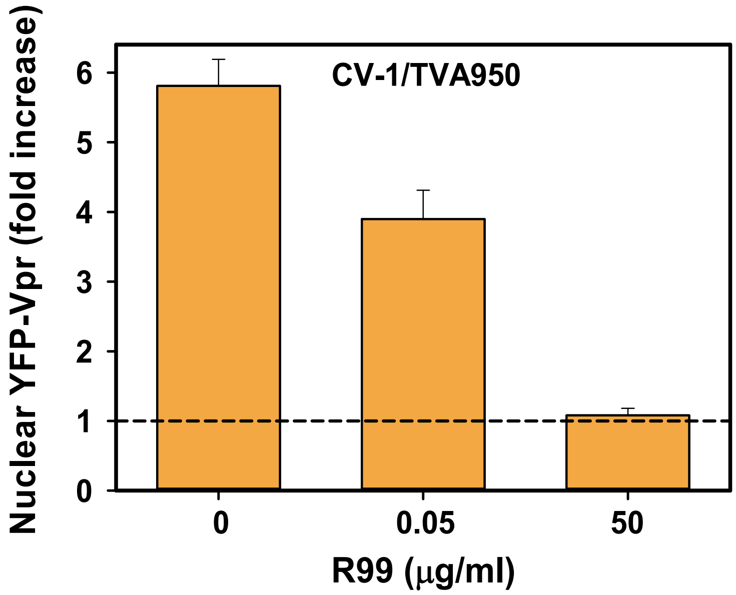
Nuclear YFP-Vpr (fold increase)
CV-1/TVA950
0
1
2
3
4
5
6
0
0.05
50
R99 (μg/ml)

Suppl. Fig. S1

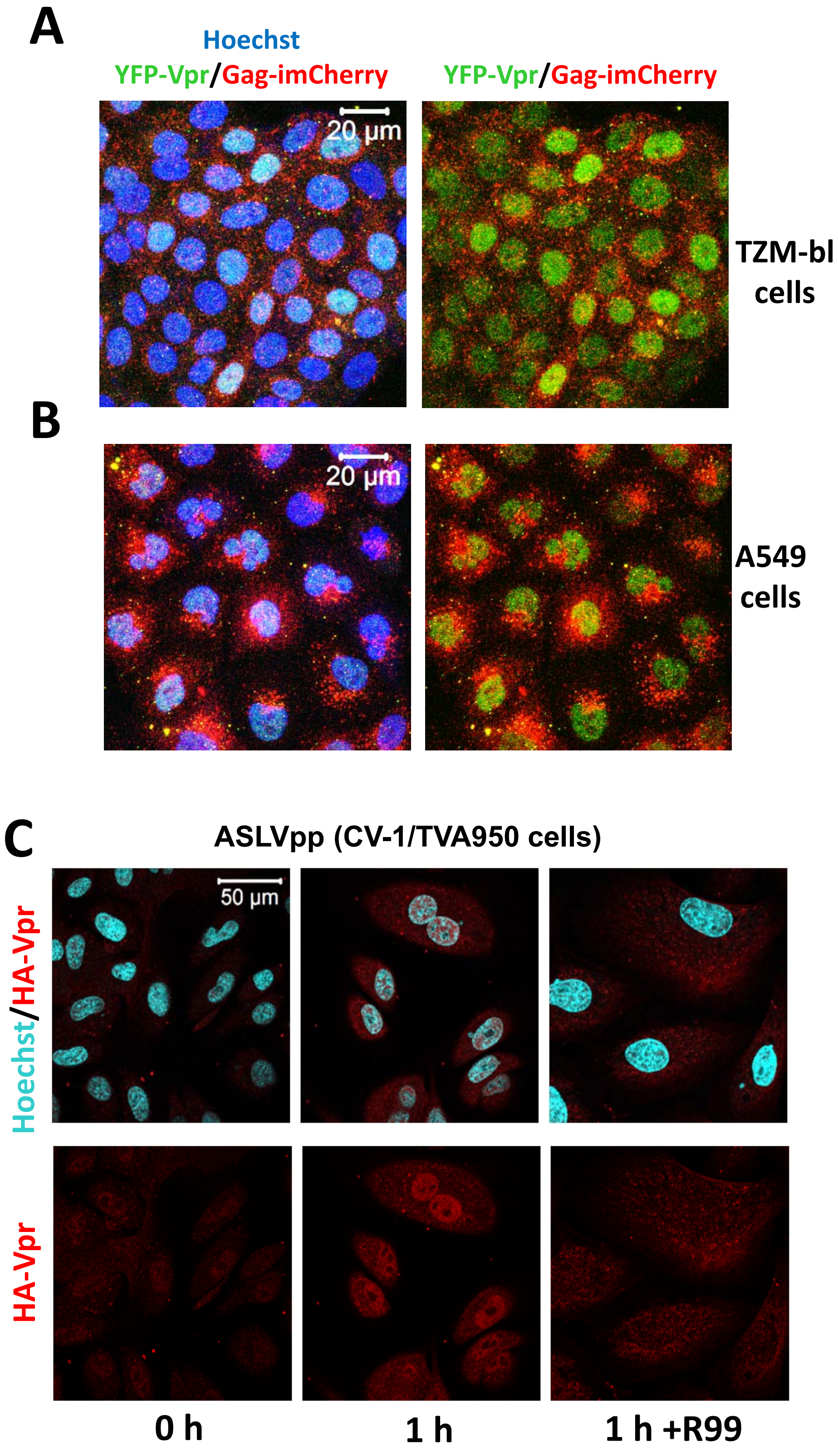
A
Hoechst
YFP-Vpr/Gag-imCherry
YFP-Vpr/Gag-imCherry
20 μm
TZM-bl cells
B
20 μm
A549 cells
C
ASLVpp (CV-1/TVA950 cells)
50 μm
Hoechst/HA-Vpr
HA-Vpr
0 h
1 h
1 h +R99

Suppl. Fig. S2

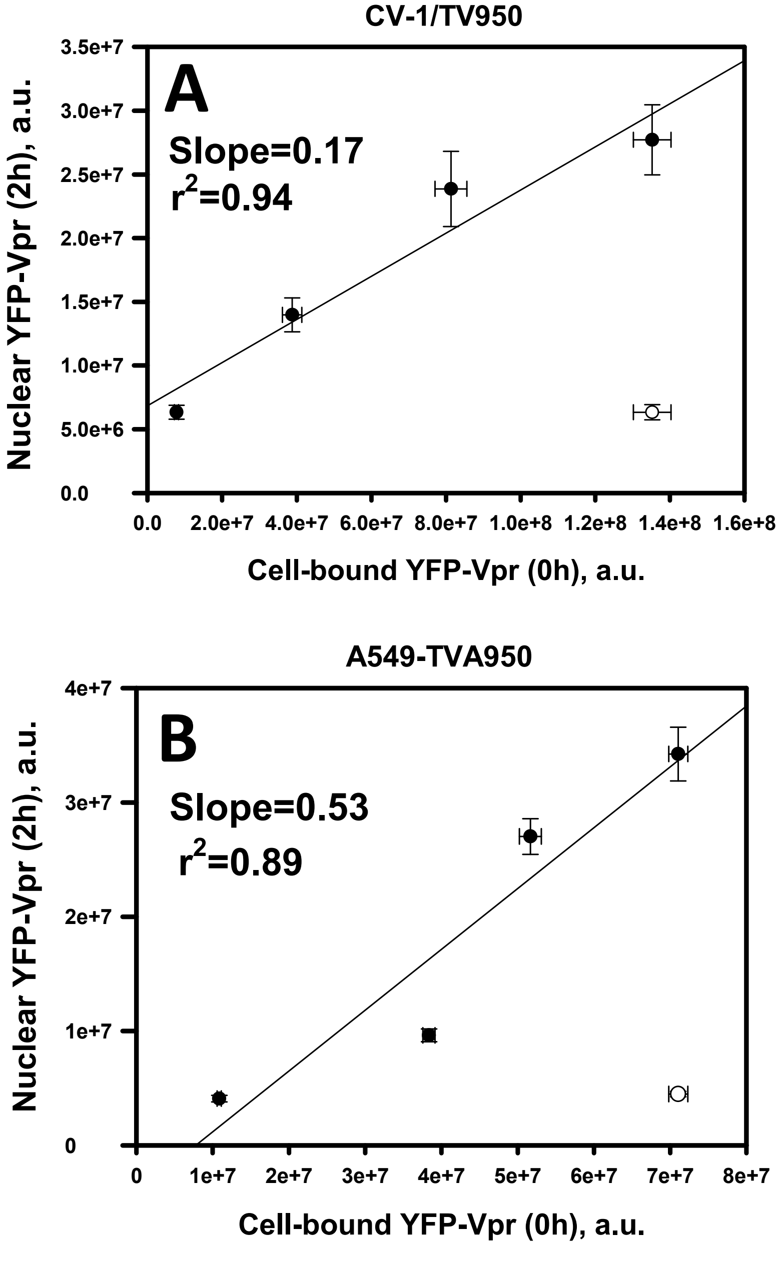
CV-1/TV950
A
Slope=0.17
r^2=0.94
Nuclear YFP-Vpr (2h), a.u.
Cell-bound YFP-Vpr (0h), a.u.
3.5e+7
3.0e+7
2.5e+7
2.0e+7
1.5e+7
1.0e+7
5.0e+6
0.0
0.0
2.0e+7
4.0e+7
6.0e+7
8.0e+7
1.0e+8
1.2e+8
1.4e+8
1.6e+8
A549-TVA950
B
Slope=0.53
r^2=0.89
Nuclear YFP-Vpr (2h), a.u.
Cell-bound YFP-Vpr (0h), a.u.
4e+7
3e+7
2e+7
1e+7
0
0
1e+7
2e+7
3e+7
4e+7
5e+7
6e+7
7e+7
8e+7

Suppl. Fig. S3

CV-1/TVA950 cells

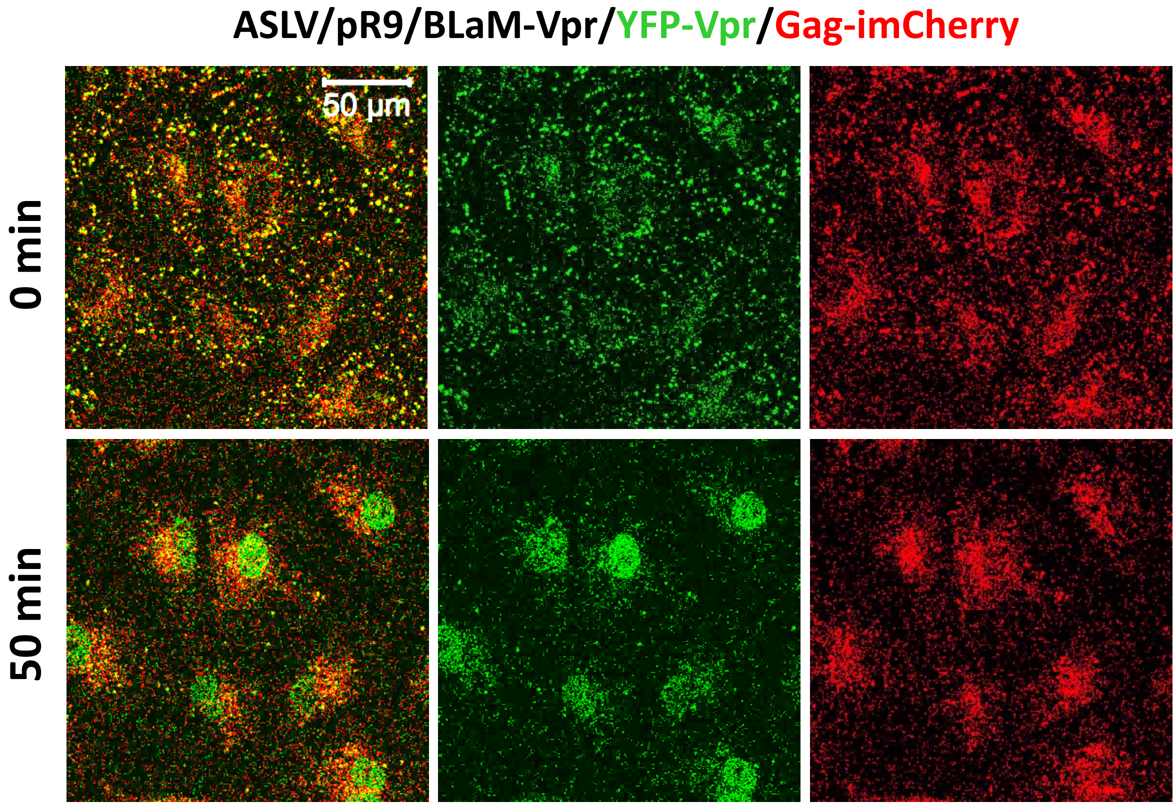

Suppl. Fig. S4

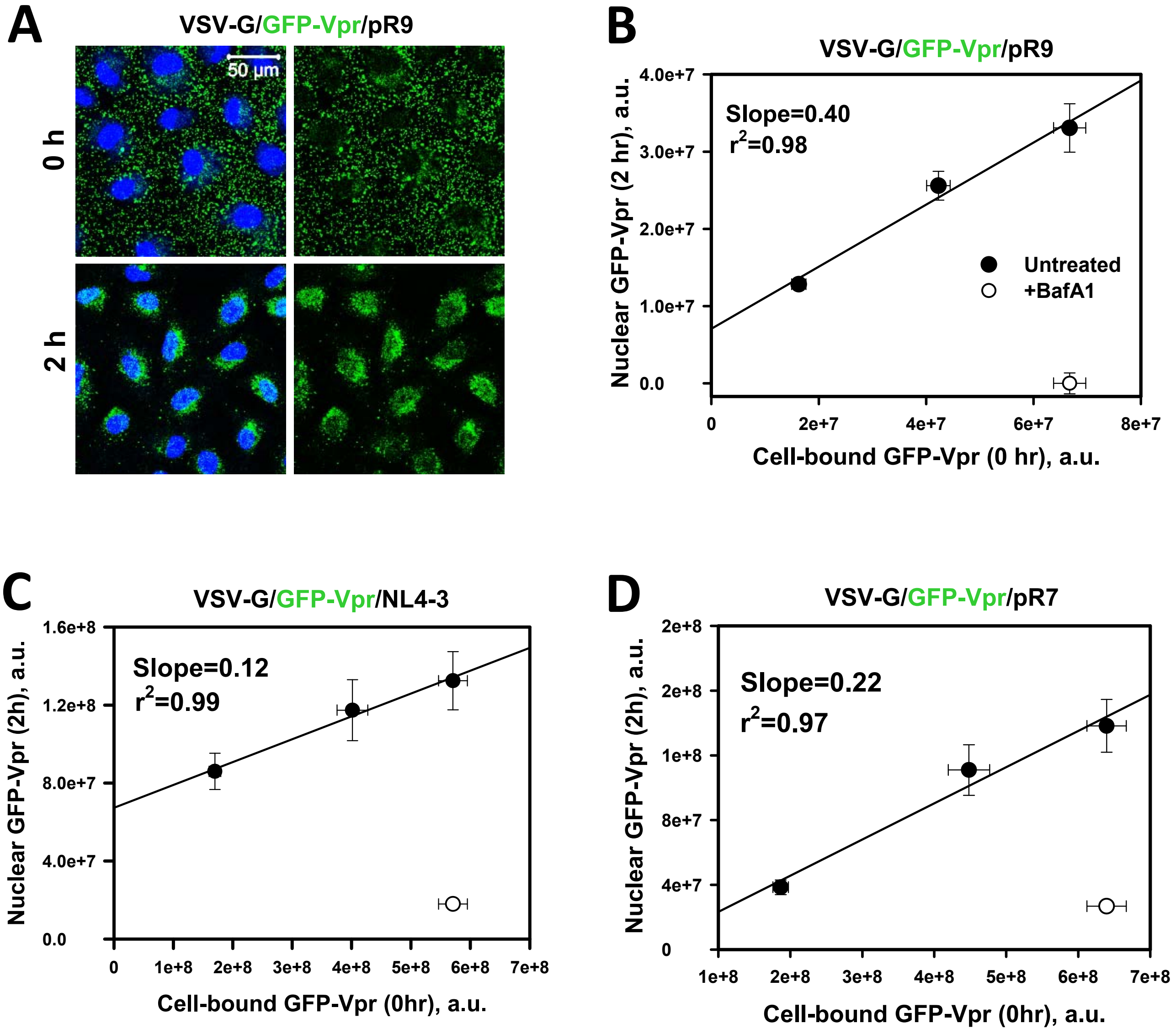

A
VSV-G/GFP-Vpr/pR9
50 µm
0 h
2 h
B
VSV-G/GFP-Vpr/pR9
Slope=0.40
r^2=0.98
Untreated
+BafA1
Nuclear GFP-Vpr (2 hr), a.u.
Cell-bound GFP-Vpr (0 hr), a.u.
C
VSV-G/GFP-Vpr/NL4-3
Slope=0.12
r^2=0.99
Nuclear GFP-Vpr (2h), a.u.
Cell-bound GFP-Vpr (0hr), a.u.
D
VSV-G/GFP-Vpr/pR7
Slope=0.22
r^2=0.97
Nuclear GFP-Vpr (2h), a.u.
Cell-bound GFP-Vpr (0hr), a.u.

Suppl. Fig. S5

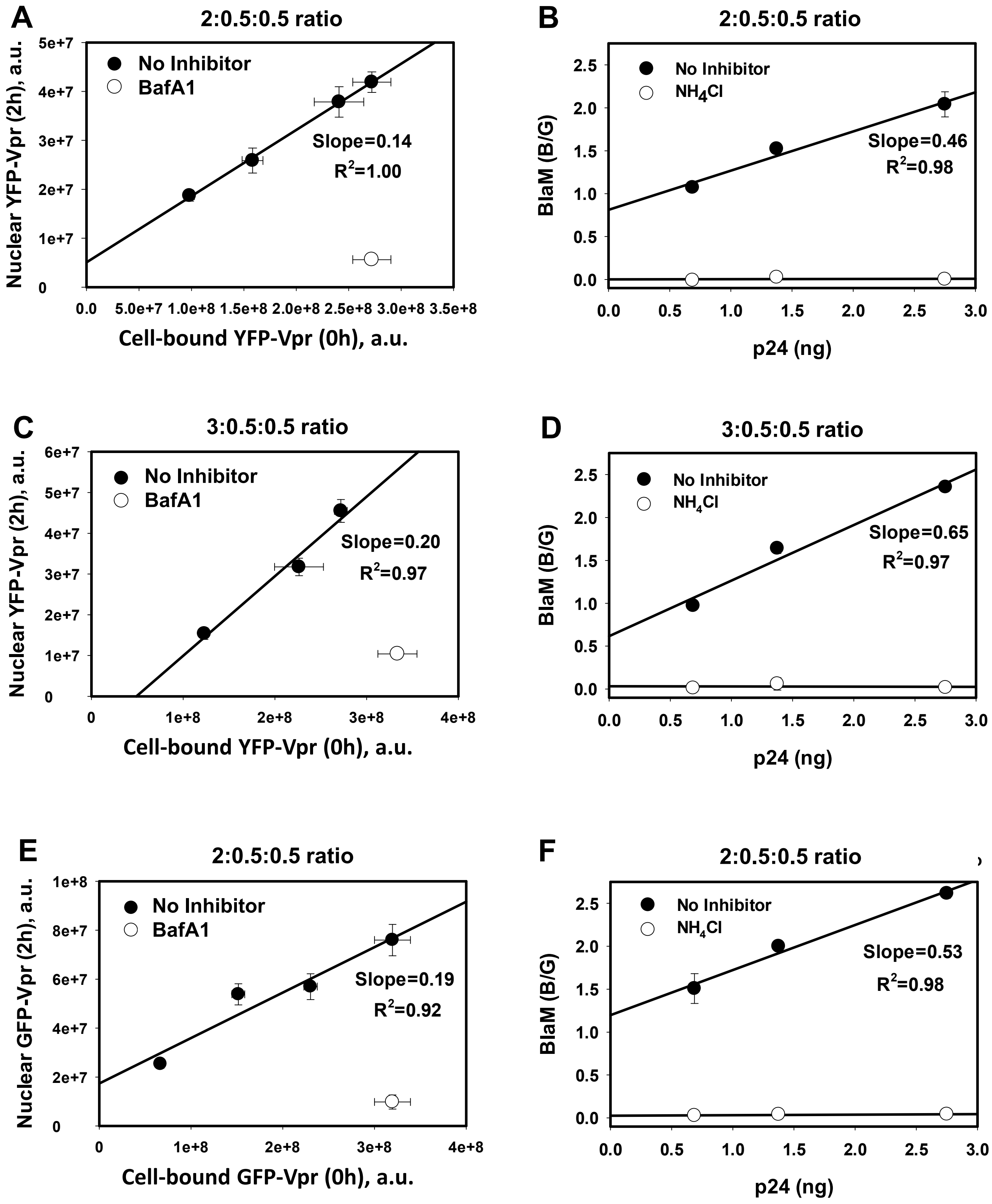
A
2:0.5:0.5 ratio
No Inhibitor
BafA1
Slope=0.14
R^2=1.00
Nuclear YFP-Vpr (2h), a.u.
Cell-bound YFP-Vpr (0h), a.u.
0
1e+7
2e+7
3e+7
4e+7
5e+7
0.0
5.0e+7
1.0e+8
1.5e+8
2.0e+8
2.5e+8
3.0e+8
3.5e+8
B
2:0.5:0.5 ratio
No Inhibitor
NH_4Cl
Slope=0.46
R^2=0.98
BlaM (B/G)
p24 (ng)
0.0
0.5
1.0
1.5
2.0
2.5
3.0
C
3:0.5:0.5 ratio
No Inhibitor
BafA1
Slope=0.20
R^2=0.97
Nuclear YFP-Vpr (2h), a.u.
Cell-bound YFP-Vpr (0h), a.u.
0
1e+7
2e+7
3e+7
4e+7
5e+7
6e+7
1e+8
2e+8
3e+8
4e+8
D
3:0.5:0.5 ratio
No Inhibitor
NH_4Cl
Slope=0.65
R^2=0.97
BlaM (B/G)
p24 (ng)
E
2:0.5:0.5 ratio
No Inhibitor
BafA1
Slope=0.19
R^2=0.92
Nuclear GFP-Vpr (2h), a.u.
Cell-bound GFP-Vpr (0h), a.u.
0
2e+7
4e+7
6e+7
8e+7
1e+8
2e+8
3e+8
4e+8
F
2:0.5:0.5 ratio
No Inhibitor
NH_4Cl
Slope=0.53
R^2=0.98
BlaM (B/G)
p24 (ng)

Suppl. Fig. S6

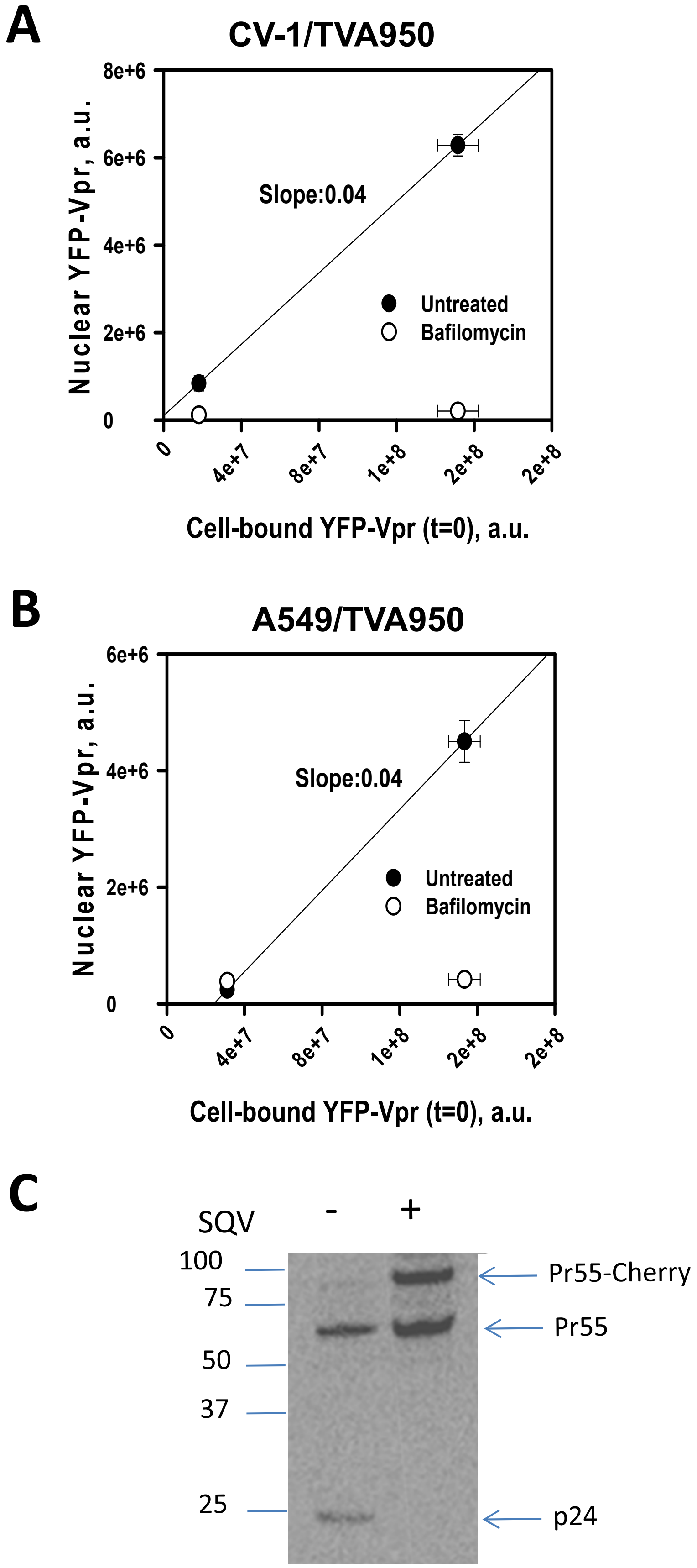
A
CV-1/TVA950
Nuclear YFP-Vpr, a.u.
8e+6
6e+6
4e+6
2e+6
0
Slope:0.04
Untreated
Bafilomycin
0
4e+7
8e+7
1e+8
2e+8
2e+8
Cell-bound YFP-Vpr (t=0), a.u.
B
A549/TVA950
Nuclear YFP-Vpr, a.u.
6e+6
4e+6
2e+6
0
Slope:0.04
Untreated
Bafilomycin
0
4e+7
8e+7
1e+8
2e+8
2e+8
Cell-bound YFP-Vpr (t=0), a.u.
C
SQV
-
+
100
75
50
37
25
Pr55-Cherry
Pr55
p24

Suppl. Fig. S7

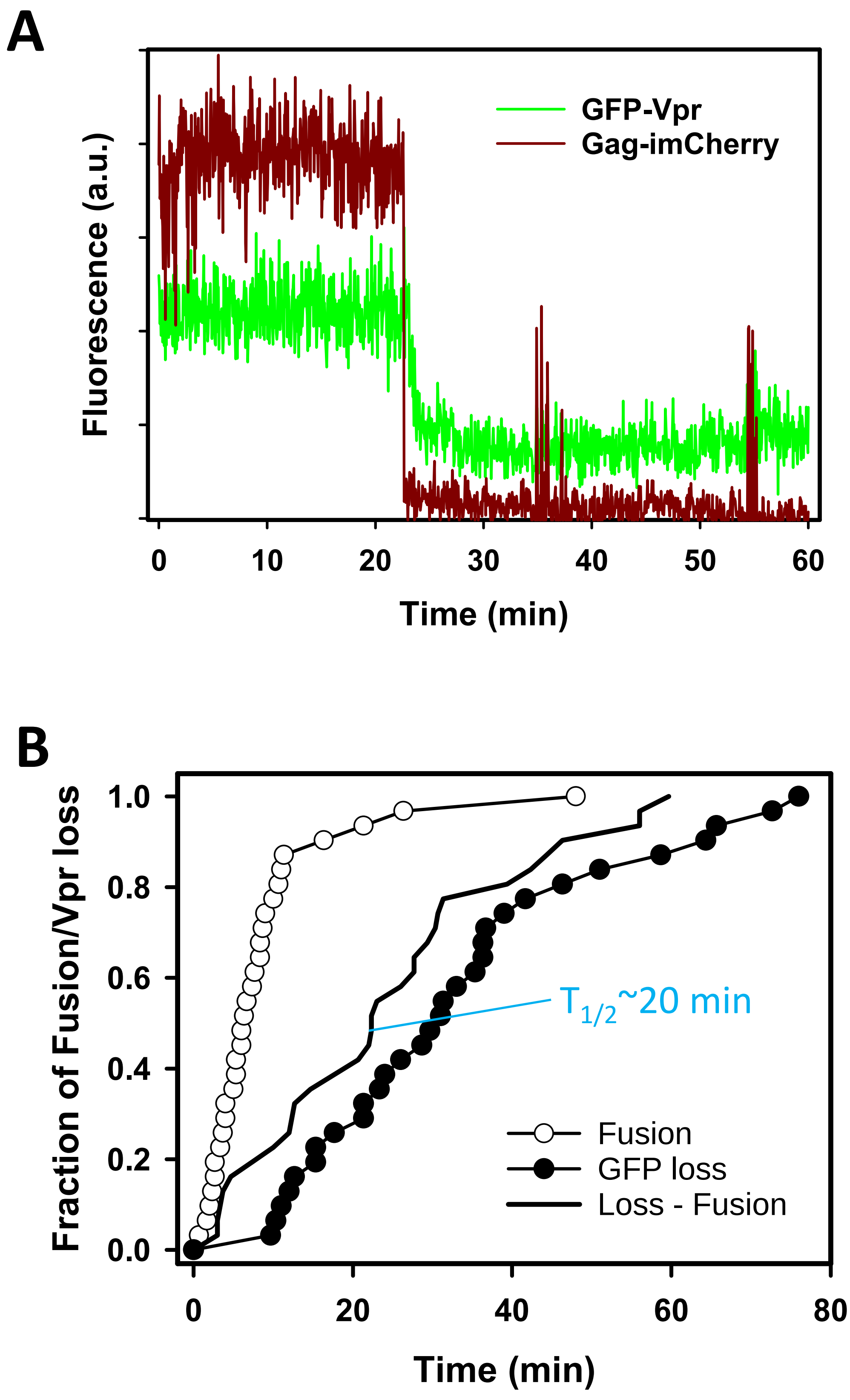
A
GFP-Vpr
Gag-imCherry
Fluorescence (a.u.)
0
10
20
30
40
50
60
Time (min)
B
Fraction of Fusion/Vpr loss
1.0
0.8
0.6
0.4
0.2
0.0
0
20
40
60
80
Time (min)
T1/2~20 min
Fusion
GFP loss
Loss - Fusion

Suppl. Fig. S8

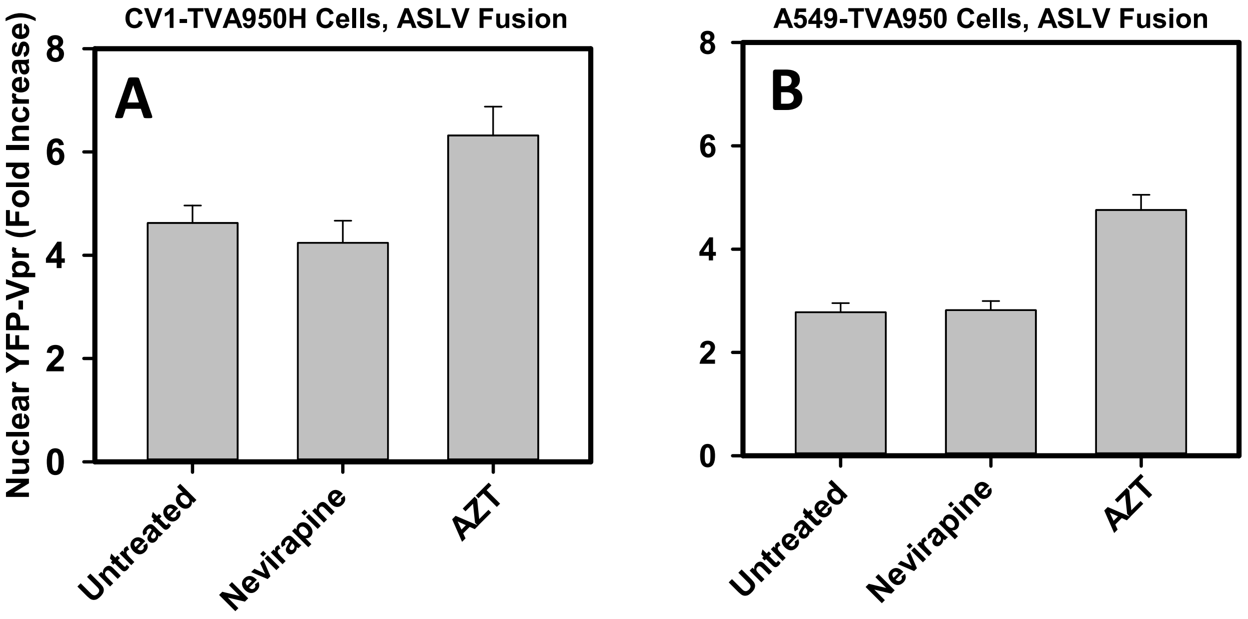
CV1-TVA950H Cells, ASLV Fusion
A
Nuclear YFP-Vpr (Fold Increase)
0
2
4
6
8
Untreated
Nevirapine
AZT
A549-TVA950 Cells, ASLV Fusion
B
0
2
4
6
8
Untreated
Nevirapine
AZT

Suppl. Fig. S9

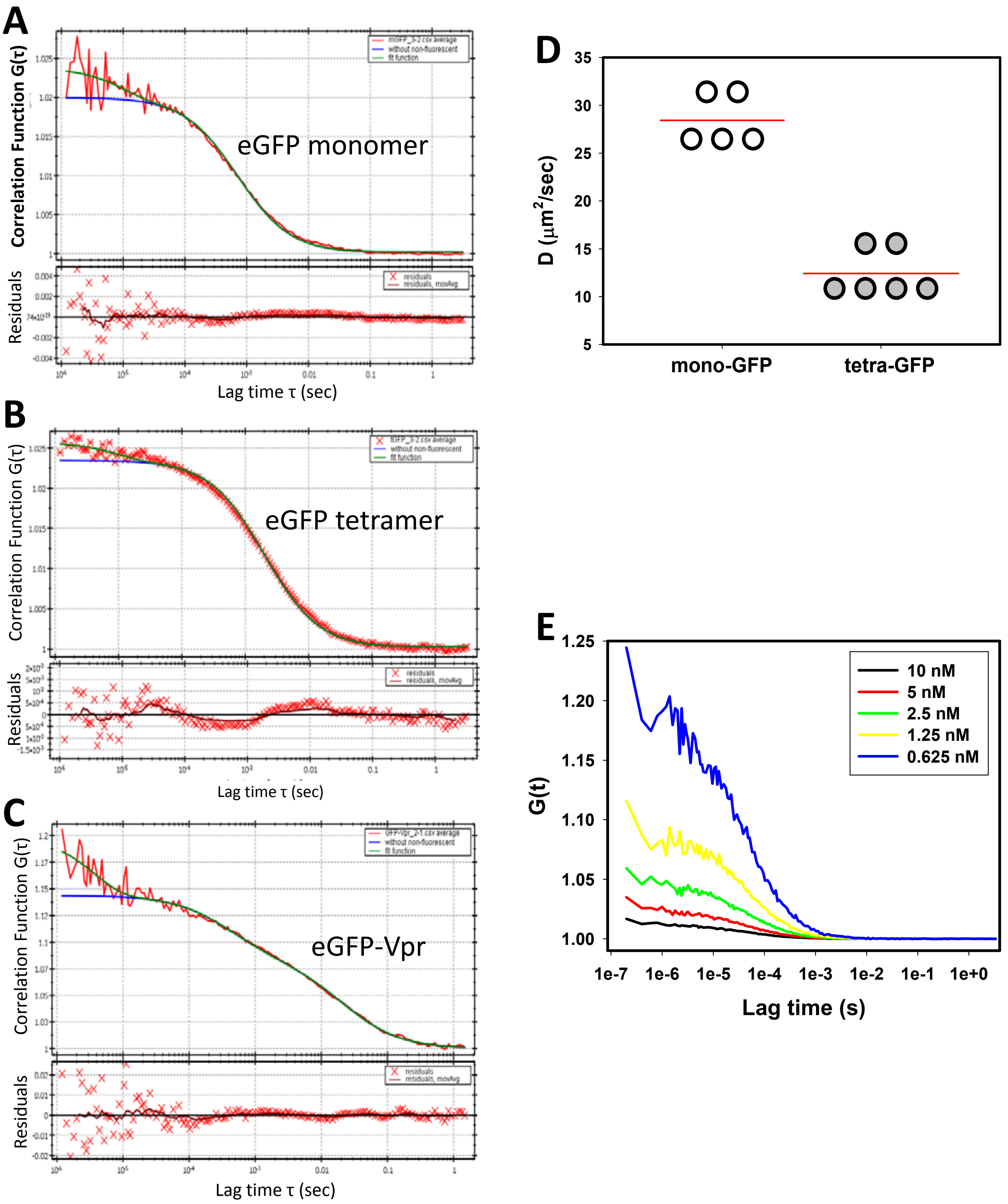

Suppl. Fig. S10
